# *First-in-Man* Open Clinical Trial of a Combined rdESAT-6 and rCFP-10 Tuberculosis Specific Skin Test Reagent

**DOI:** 10.1371/journal.pone.0011277

**Published:** 2010-06-25

**Authors:** Winnie Bergstedt, Pernille N. Tingskov, Birgit Thierry-Carstensen, Søren T. Hoff, Henrik Aggerbeck, Vibeke O. Thomsen, Peter Andersen, Aase B. Andersen

**Affiliations:** 1 Department of Infectious Diseases, Rigshospitalet, University of Copenhagen, Copenhagen, Denmark; 2 Department of Vaccine Development, Statens Serum Institut, Copenhagen, Denmark; 3 Department of Infectious Disease Immunology, Statens Serum Institut, Copenhagen, Denmark; 4 International Reference Laboratory of Mycobacteriology, National Centre for Antimicrobials and Infection Control, Statens Serum Institut, Copenhagen, Denmark; University of Cape Town, South Africa

## Abstract

**Background:**

Tuberculin is still the only available skin test reagent for the diagnosis of mycobacterial infection. The product has a remarkable sensitivity, but poor specificity. Previous studies, including two human phase I clinical trials, have indicated that rdESAT-6 has a potential as an improved skin test reagent. Animal studies have shown that the sensitivity may be increased by inclusion of the genetically related CFP-10 antigen in the preparation without loosing specificity.

**Methodology:**

In this study a *Lactococcus* fermented, recombinant skin test reagent consisting of a 1∶1 wt/wt of rdESAT-6 and CFP-10 was manufactured according to GMP standards and tested for the first time in 42 healthy adult volunteers. The two doses of 0.01 µg or 0.1 µg were injected intradermally by the Mantoux technique with 6 or 12 weeks interval. No serious adverse events and only mild adverse reactions were reported. The reagent elicited a positive skin test reaction after the first injection in one participant, who most likely was latently infected with *M. tuberculosis* as indicated by an appreciable IFN γ response just below the Quantiferon® cut-off level at the screening visit. None of the remaining participants in the four groups had any skin test reactions and sensitisation by the reagent could therefore be excluded.

**Conclusion:**

The investigational skin test reagent rdESAT-6 and CFP-10 appeared safe and non-sensitising in this first-in-man clinical trial in human volunteers and can now be tested in larger clinical trials involving individuals with latent *M. tuberculosis* infection or active TB disease.

**Trial Registration:**

ClinicalTrials.gov NCT00793702

## Introduction

The potential of *in vitro* based methods to detect immune reactivity towards tuberculin antigen has been widely accepted in recent years. Two commercially available assays are based on immune recognition of the *Mycobacterium tuberculosis* specific antigens ESAT-6, CFP-10 and in one of the assays also TB-7.7. Immune T lymphocytes release interferon-γIFNγ after presentation to the *M. tuberculosis* antigens and subsequent ELISA or EliSpot assays quantify the cytokine. An accepted acronym for such tests is *IGRA*: Interferon-Gamma-Release-Assays [1], [2]. IGRAs have several advantages compared to the conventional Tuberculin skin test (TST) especially increased specificity. The antigens included in the IGRAs have been selected based on their absence in most *non-tuberculous* mycobacteria and in the vaccine strain *M. bovis* BCG altogether limiting “false positive” reactions [3], [4]. However, in many parts of the world laboratory facilities required to perform an IGRA are not available. Skin testing by the Mantoux technique with standardised amounts of tuberculin e.g. Purified Protein derivative (PPD) is indeed a “low-tech” procedure, which is easily performed by trained health care personnel any time during the day, any day of the week, independently of the availability of laboratory personnel or equipment [5].

Tuberculins like PPD are composed of a crude mixture of heat-denatured proteins derived from cultures of *M. tuberculosis*. The purification process ensures the removal of lipids and cell walls but the exact quantity of the individual antigens is not known and dosing is based on a biological assay measuring cellular infiltration (i.e. induration) in the skin of immune guinea pigs as read-out. It is therefore not surprising that other mycobacteriel species (like the BCG vaccine strain) possessing cross-reactive antigens can induce a reaction. Also, as skin testing involves intradermal injection of antigen into the person to be tested, repeated testing may cause a sensitisation reaction, depending on the time interval between testing [6], [7]. The identification of *M. tuberculosis* specific antigens has prompted us to explore the potential of such antigens as improved, next-generation, tuberculosis (TB) skin test reagents with higher specificity, retained diagnostic sensitivity and low sensitising properties. We have previously reported preliminary results using rdESAT-6 as a skin test reagent in healthy adult volunteers and in cured TB patients [8], [9]. In the present study we investigated increasing doses of a potential skin test reagent composed of the species-specific rCFP10 and rdESAT6 antigens in a first-in-man, phase I, clinical trial in healthy, adult volunteers.

## Methods

### Study design

The study was designed as an open, phase I clinical trial and conducted at the Department of Infectious Diseases at Rigshospitalet (a third level referral national hospital) in Copenhagen, Denmark. The study included 42 non-black volunteers ≥18 years of age from December 2008 to June 2009. Non-black volunteers were selected to obtain the optimal conditions for an accurate visual evaluation of the size of the skin test reactions (induration and/or redness). The volunteers were mainly recruited by advertising in newspapers and at bulletin boards at adjacent educational institutions. All gave written informed consent prior to inclusion and were healthy according to medical examination, medical history and laboratory tests. The most important exclusion criteria were: previous history of TB or known contact to a person with active TB, positive IFN γ response by QuantiFERON® TB-Gold In-Tube test (QFT-IT) at inclusion, volunteering in former trials assessing rdESAT-6, immunosuppressive treatment, congenital and/or acquired immune deficiency, vaccination with live vaccines within the last 6 months, ongoing viral and/or bacterial infection, severe skin disease and pregnancy. The protocol for this trial and supporting CONSORT checklist are available as supporting information; see Checklist S1 and Protocol S1.

#### Allocation in study groups

**Figure 1:**
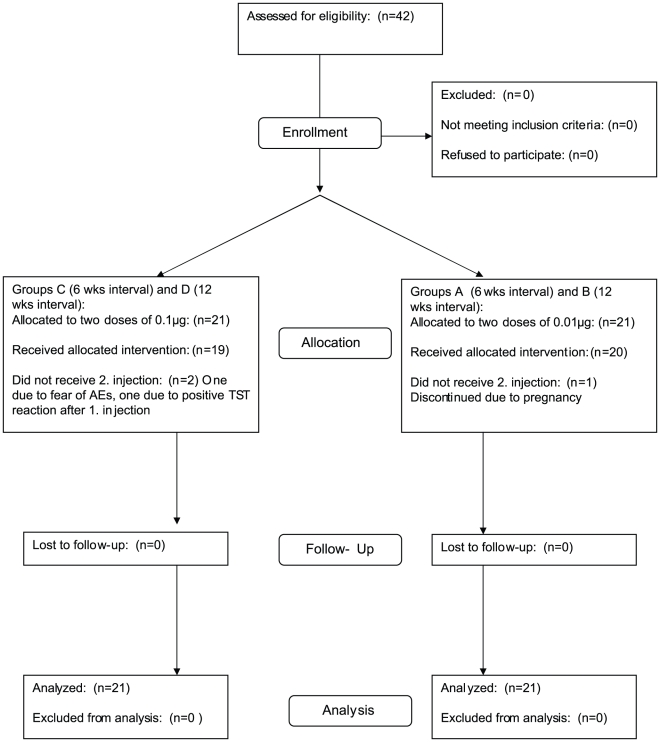
Flow diagram of study design. Participants were screened from −28 to −3 days before inclusion. The injections were given at day 0 and day 42 or on day 0 and day 84. Group A and B received 0.01 µg of the investigational skin test antigen. Group C and D received 0.1 µg. All volunteers completed a final follow-up visit 28 days after the last injection.

The trial was designed to include 5 groups with 10 volunteers in each group. Group A and B volunteers received two intradermal doses of 0.01 µg rdESAT-6 and rCFP-10 (w/w ratio 1∶1), with time intervals of 6 weeks for Group A and 12 weeks for Group B. Group C and D volunteers received two intradermal doses of 0.1 µg of rdESAT-6 and rCFP-10, with a time interval between the two doses of 6 weeks for Group C and 12 weeks for Group D. Group E was scheduled to receive only one dose of rdESAT-6 and rCFP-10 of 1.0 µg. However, due to opacities observed visually in the vials containing the highest concentration of the experimental skin test reagent, this group was never recruited or initiated. A flow-chart describing the experimental design is depicted in Figure 1. After being found eligible for the trial at the inclusion visit the volunteers were allocated to the first (not yet occupied) study group with an acceptable time schedule in the order A, B, C and D.

#### Skin testing procedure

The rdESAT-6 and rCFP-10 reagent was injected by the Mantoux technique with a short-bevelled sterile needle sized 0.45×10 mm (26 Gauge) in the dorsal aspect of the right (first injection) and left (second injection) forearm, respectively. Observation of a small papule indicated correct injection technique. During the first hour after the injection, the volunteers were monitored closely for the occurrence of immediate adverse reactions. For safety reasons there was at least 1 hour between the injections of different volunteers. Follow-up visits took place 72 hours after administration of the first dose and 72 hours and 28 days after administration of the second dose. Digital photographs were taken of all injection sites at the 72 hours visit. The diameters of the skin indurations and/or redness of the injection sites were measured transversally to the long axis of the forearm 72 hours after both the first and second injection. “Redness” was defined as visible red or pink discoloration of the skin around the injection site and erythema ≥20 mm was regarded as a local adverse reaction. “Induration” was measured by palpation from lateral to central on all sites using a ball pen. Induration ≥6 mm was regarded as a possible sensitisation reaction after the second injection. A skin reaction after the first injection was regarded as a sign of latent *M. tuberculosis* infection or an adverse reaction.

#### QFT-IT

In vitro IFN γ responses were measured in blood samples taken at the screening visit, prior to administration of the second rdESAT-6 and rCFP-10 dose and at termination visit. An IFN γ response ≥0.35 IU/ml at screening was regarded as possible *M. tuberculosis* infection. Responses occurring after the second injection were regarded as possible sensitisation reactions. The result of the second QFT-IT was not known when the second Mantoux test with rdESAT-6 and rCFP-10 was administered. In case of possible sensitisation reactions, specific IFN γ responses were measured by *in vitro* stimulation of Purified Blood Mononucleated Cells (PBMC) from an extra blood sample with a selected panel of antigens (see below) and an additional QFT-IT test was performed.

### Adverse events/reactions

All systemic and local adverse events occurring in the time period between the first test administration and 28 days after the second test administration were recorded. An adverse event was defined as any untoward medical occurrence in a volunteer exposed to the rdESAT-6 and rCFP-10 skin test regardless of causal relationship with the product. Causality was assessed using the terms: not related, possible, probable or certain. The intensity was assessed using the terms: mild (easily tolerated), moderate (interfere with daily activities) or severe (prevent normal activity). Seriousness was assessed using standard terms. The volunteers were asked to fill in diaries between the trial visits to keep track of symptoms, temperature measurements and concomitant medication. All adverse events/reactions were coded using Medical Dictionary for Regulatory Activities (MedDRA) preferred terms.

### The investigational rdESAT-6 and rCFP-10 skin test reagent

The investigational skin test reagent rdESAT-6 and rCFP-10 was manufactured at SSI, Denmark following Good Manufacturing Practice (GMP) standards. The product is composed of recombinant versions of the two *M. tuberculosis* derived antigens ESAT-6 and CFP-10 cloned and expressed in *Lactococcus lactis* [7], [10]. rdESAT-6 is a dimeric version of ESAT-6 with 5 amino acids extra at the N-terminal end of the protein and three amino acids separating the two monomers. The rCFP-10 protein also has an N-terminal extension of four amino acids. The two purified proteins were mixed in a weight ratio of 1∶1 in a vehicle of PBS with 0.01% Polysorbate 20® and aliquots dispensed into vials for storage at +2°C to +8°C. ESAT-6 and CFP-10 form 1∶1 complex in nature [10].

### In-house Interferon-Gamma Release Assay

Frozen human PBMCs were thawed and 2×10^5^ cells were incubated in triplicate for five days at 37°C with 5% CO_2_ with either culture medium alone, Staphylococcal Enterotoxin B at 1 ug/mL (Sigma cat. number S-4881, Sigma-Aldrich, DK) or three different peptide mixtures at 2.5 ug/ml/peptide each. The peptide mixtures were all spanning the entire protein of selected *M. tuberculosis* proteins TB 10.4 (9 peptides, 18-mer, 8 overlap; JPT peptide, Germany) [11], ESAT-6 (13 peptides, 15-mer, 9 overlap; JPT peptides, FRG) or CFP-10 (15 peptides, 15-mer, 9 overlap; Genescript, USA). Subsequently IFN γ was measured by ELISA in the supernatants with a standard sandwich enzyme-linked immunosorbent assay technique with a commercially available pair of monoclonal antibodies (product # M700A and M701B, Thermo Fisher Scientific Inc.) and used according to the manufacturer's instruction. Recombinant IFN γ (product # RIFNG50, Thermo Fisher Scientific Inc.) was used as a standard. Cytokine levels are given as pictogram of protein per millilitre of supernatant (pg/mL).

### Data safety monitoring board

A data safety monitoring board (DSMB) was established consisting of three independent senior clinicians: two from other Danish hospitals and one from a Dutch hospital. The principal investigator was responsible for the safety of the volunteers. However, DSMB members could be consulted for expert advice.

### Sample size determination

The sample size was primarily based on practical and safety issues and not statistical considerations. However, assuming the risk of sensitisation defined as induration >6mm or IFN g >0.35 IU/mL as measured in the QuantiFERON TB Gold analysis after the second injection, increases with dose and for a fixed dose of the experimental skintest reagent is higher if the two doses are given with a 6 week interval than with a 12 week interval. If none of the 40 participants in the groups show evidence of sensitisation, then not only the 10 participants in that particular group but also those receiving a higher dose and or shorter injection interval can be taken into consideration in a conservative calculation of a one-sided upper 95% CI for the risk of sensitisation. With an effective sample size n, the conservative one-sided upper 95% CI was calculated as [0, 1−(1−0.95) ^(1/n)^] resulting in a CI for sensitisation in the groups receiving the low dose (0.01µg) of 0.00 to 0.07 with an effective sample size of 40 and for the high dose groups receiving 0.1µg the CI was 0.00 to 0.14 with an effective sample size of 20.

### Ethical issues

The study was sponsored by SSI, Copenhagen, DK (Trial code TESEC-01, EudraCT no.: 2008-001489-96) and conducted according to “Note for guidance on good clinical practice”, CPMP/ICH/135/95, ICH topic E6 and CPMP/ICH/377/95 E 2A. The funder had no role in study design, data collection and analysis, decision to publish, or preparation of the manuscript. The study was approved by the Danish Medical Ethics Committee System (no: H-A-2008-105) and the Danish Data Protection Agency (no: 2001-54-0877). The study was inspected and approved with minor comments by the Danish Medicines Agency before initiation (approval no: 2612-3851). The trial was filed in the NIH clinical trials database: NCT00793702.

## Results

42 volunteers (12 (29%) males were screened and all met the inclusion criteria and were enrolled in the study. The mean age was 36 years (SD 11,4 years), 40 were Danish and two volunteers originated from another European country. Twenty (48%) had been BCG vaccinated and 12 (29%) had previously had a TST. One volunteer was excluded before the second injection was given due to a positive pregnancy test. The volunteers were allocated by order of appearance into four groups (Groups A to D) that differed by the time intervals between the two injections and the dose level of rdESAT-6 and rCFP-10 (Figure 1).

### Group A

Group A included 7 women and 4 men, mean age 40 years (SD 11 years). Eight volunteers had a history of BCG-vaccination and three reported a TST at some occasion, but could not recollect the exact time. One person did not remember his BCG status. None of the volunteers in Group A showed any positive skin reactions or IFN γ responses as measured by QFT-IT following the two injections of 0.01 µg of rdESAT-6 and rCFP-10 with 6 weeks interval.

### Group B

Group B included 7 women and 3 men, mean age 33 years (SD 11 years).

Three volunteers had previously been BCG-vaccinated and two had been skin tested before inclusion in the study, but were not able to recall the exact time. None of the volunteers in group B showed any positive reaction, neither skin reactions nor IFN γ responses by QFT-IT following two injections of 0.01 µg of rdESAT-6 and rCFP-10 with 12 weeks interval.

### Group C

**Figure 2:**
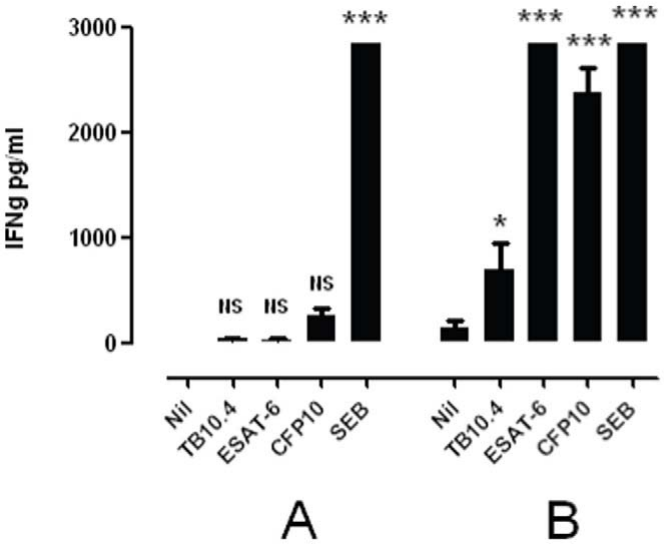
In vitro IFN γ responses to selected antigens. PBMCs from two volunteers were tested for in vitro IFN γ responses to selected *M. tuberculosis* antigens. A:volunteer from group C, TST negative, QFT-IT positive after 1. injection, but negative after 2. injection and at day 28. B: volunteer from group D, TST and QFT-IT positive after 1. injection and hereafter excluded. PBMCs were stimulated for five days with culture medium only (Nil), TB10.4 peptide mixture (TB10.4), ESAT-6 peptide mixture (ESAT-6), CFP10 peptide mixture (CFP10) or Staphylococcal Enterotoxin B (SEB) as a positive control. Subsequently, the supernatants were tested for IFN γ content by ELISA. Bars indicate the mean concentrations of IFN γ in pg/mL of triplicate wells. Error bars indicate the Standard Error of Mean. For each subject, the means of the peptide- and SEB stimulated wells were compared to the mean of the corresponding un-stimulated (Nil) wells by one-way analysis of variance with Bonferoni post-test correction. ***: P<0.001, *:P<0.05, NS: Not Significant (P>0.05).

Group C included 7 women and 3 men, mean age 34 years (SD 12 years). Five volunteers reported previous BCG-vaccination and three had been tuberculin skin tested at some unknown point of time before inclusion in the study. One volunteer was unaware of his vaccination and TST status. No positive skin reactions occurred following the two intradermal injections of 0.1 µg of rdESAT-6 and rCFP-10 with 6 weeks interval. However, one volunteer, a 23-year-old man, who had neither been BCG vaccinated or skin tested previously and had a negative IFN γ response at screening of 0.02 IU/ml, showed a positive IFN γ response by QFT-IT of >10.0 IU/ml prior to administration of the second injection, decreasing to 0.02 IU/ml 28 days later. At this point in time an additional in-house IGRA was performed showing no response to ESAT-6 or CFP-10 peptide stimulation (Figure 2A). This finding confirmed the negative second QFT-IT test result. He had no visible or palpable skin reaction. None of the remaining volunteers in this group developed a positive IFN γ response.

### Group D

Group D included 9 women and 2 men, mean age 37 years (SD 12 years). Four were previously BCG-vaccinated and four had a history of a TST. Two persons were unaware of their status. One 55-year-old woman in this group withdrew from the trial just before the second injection due to fear of side effects. A 34-year-old woman developed a 20 mm induration and redness at the injection site 72 hours after administration of the first dose of rdESAT-6 and rCFP-10. She was not BCG-vaccinated, but had received a TST 5 years previously. Furthermore, she had a positive QFT-IT IFN γ response of 1.57 IU/ml 96 hour after the first injection. Noteworthy, this volunteer had the highest IFN γ response at the time of the screening visit at 0.24 IU/ml (cut off point: 0.35 UI/ml). An additional in-house IGRA was performed confirming the positive IFN γresponse to both ESAT-6 and CFP-10 peptide stimulation as to peptides from TB10.4 protein: an *M. tuberculosis* protein not present in the skin-test reagent (Figure 2B). This participant was hereafter excluded from the study and did not receive the second injection. None of the remaining 9 volunteers showed any positive skin test or IFN γ reactions.

#### Sensitisation results

39 volunteers received two injections and none of them experienced induration, erythema or a positive QFT-IT INF γ result 28 days after the second skin test (CI 0.1 µg/0.1mL, short interval: 0.00–0.26; CI 0.1 µg/0.1mL, long interval: 0.00–0.19; total CI: 0.00–0.07).

### Adverse reactions

No serious adverse events were reported. Twenty-nine of the 42 volunteers reported at least one adverse event: related or unrelated to the skin test administration, but all of mild intensity. Twelve of 42 volunteers reported adverse reactions where a causal relationship to the skin test administration could not be ruled out. Eleven reactions were noted following the first injection and five after the second injection (Table 1).

**Table 1:** 

Adverse reactions	Group A	Group B	Group C	Group D	All groups
	N = 11	N = 10	N = 10	N = 11	N = 42
	n	n	n	n	n
Influenza like illness	2			1	7.1
Pruritus and pain	1				2.4
Skin hypopigmentation		1			2.4
Injection site pain		1			2.4
Headache		1	1	1	7.1
Application site bruising					2.4
Injection site discomfort and nausea				1	2.4
Body temperature changes^1^				1	2.4
Abnormal standard blood safety tests^2^	none	none	none	none	0

N = number of subjects in group.

n = number of subjects in group experiencing adverse reaction.

% = percentage of subjects in trial experiencing adverse reaction.

1 Recorded body temperature of 38.1°C.

2 Blood samples at final visit for: Leukocytes (WBC) (eosinophils, basophils, neutrophils, lymphocytes,and monocytes), Platelets (Plts),Haemoglobin (Hb), C-reactive protein (CRP), Glucose - random (GLU), Total bilirubin, Aspartate transferase (AST), Alanine transferase (ALT), Albumin (Alb), Creatinine, Potassium (K+), Sodium (Na+) at Visits 1 and 6. HIV, HBV and HCV only at Visit 1).

## Discussion

In the process of developing a more specific and sensitive TB skin test reagent, the recombinant dimeric version of ESAT-6 has already been assessed in human phase I clinical trials involving both healthy volunteers and TB patients [8], [9]. Animal studies have shown that combining ESAT-6 with the genetically related CFP-10 protein increased sensitivity of the product without hampering specificity [12]. Human *in vitro* studies have supported that rCFP-10 can discriminate TB patients from BCG vaccinated, healthy individuals [12].

This study describes the first *in vivo* experience with rCFP-10 as a skin test reagent in humans. The study assessed two aspects: the safety in a small scale, first-in-man study in healthy volunteers and the potential risk of sensitisation induced by repeated doses of the investigational skin test antigen. No serious adverse events were observed during the study. Only mild and predictable events were recorded. Positive QFT-IT IFN γ reactions were induced in two volunteers. In one case it was associated with a positive skin test reaction after the first dose of antigen was injected. The participant in question works as a nurse and was TST and QFT-IT negative in 2004. She was never BCG vaccinated; she had travelled on several occasions to TB high endemic countries for recreational purposes but was not aware of exposure to TB patients. Retrospectively it was noted that this person had a rather high base-line QFT-IT IFN γ production of 0.24 IU/ml. This is below the QFT-IT test cut-off and is reported as a negative result, but clearly higher than the rest of the study group (data not shown). The most likely explanation is that this volunteer was in fact latently infected with *M. tuberculosis* and the intradermal injection of rdESAT-6 and rCFP-10 efficiently boosted her memory T lymphocytes. The additional *in vitro* IGRA result supports this hypothesis as she also recognised another *M. tuberculosis* antigen besides ESAT-6 and CFP10. Sensitisation due to a TST with PPD five year previously is unlikely [7], [13]. Clinical examination and follow-up exhibited no signs of or sequelae from TB disease. Another theoretical consideration could be exposure to and sensitisation by *M. kansasii* or *M. marinum*: two environmental mycobaterial species known to possess ESAT-6 and CFP-10 sensitising abilities [14]. However, the clinical history of this volunteer did not support this as a possibility as she had never suffered from pulmonary symptoms or had wound following exposure to *M. marinum* infected water like e.g. fresh-water aquariums.

One volunteer from Group C reacted positive in the QFT-IT test 6 weeks after the first injection of a 0.1 µg dose. This subject did not exhibit a positive skin reaction neither after the first nor the second injection. He converted to being QFT-IT IFN γ negative in a follow-up test 4 weeks after the second injection and an additional in vitro IGRA confirmed the follow-up negative QFT-IT test result. The investigational skin test may have induced a transitory immune reaction, which did not influence the skin test result and appeared not to be boosted by the second injection. A laboratory error in the IFN γ detection is a trivial explanation that cannot be ruled out. However, we have not been able to trace a potential source of such error.

The risk of sensitisation after repeated rdESAT-6 skin testing in 30 healthy adults has previously been reported as low although one person did develop a positive skin reaction after the second injection of 0.1µg rdESAT-6 given 28 days after the second injection [9]. In the present study the time interval between the doses were increased and the antigen doses were lower. The groups receiving the highest dose was given a total of 0.1 µg i.e. 0.05 µg of each of the two antigens which is only half the amount given in the previous study. The frequency of adverse reactions was lower in the present study compared to the previous study, which may be ascribed to the reduced dose. However, larger studies are required to give a true impression of the adverse reaction profile. In conclusion, the combination of rdESAT-6 with rCFP-10 in a novel skin test reagent appeared safe in this small-scale first-in-man clinical trial. The product itself did not sensitise non-immune healthy individuals. Larger clinical trials in relevant patient groups or individuals exposed to TB patients are therefore now being planned.

## Supporting Information

Protocol S1 (3.08 MB PDF)

Checklist S1 (0.19 MB DOC)
