# Supplementary material for: First-in-Man Open Clinical Trial of a Combined rdESAT-6 and rCFP-10 Tuberculosis Specific Skin Test Reagent
Source: PLoS One. 2010 Jun 25;5(6):e11277. doi: 10.1371/journal.pone.0011277 (PMC2892483; doi:10.1371/journal.pone.0011277)
Supplement: Protocol S1 — (3.08 MB PDF) [file pone.0011277.s001.pdf]

PROTOCOL

Final Version

Title:

An open phase I clinical trial on the safety and the risk of sensitisation by escalating doses and repeated injections of the rdESAT-6 + rCFP-10 skin test reagent following intradermal administration to healthy adults

EUDRACT No.: 2008-001489-96

Trial code: TESEC-01

Trial phase: I

Study Director:
Pernille Nyholm Tingskov
Department of Vaccine Development
Statens Serum Institut
Denmark

Confidentiality:
This document contains confidential information. The contents may not be used, divulged or published without prior written consent of Statens Serum Institut. This information cannot be used for any other purpose than the conduct and evaluation of the clinical investigation by the investigator(s), regulatory authorities and members of the ethics committee.

1. Synopsis

**Name of sponsor:**<br>Statens Serum Institut		
**Name of finished product:**<br>rdESAT-6 + rCFP-10 skin test		
**Names of active ingredient:s (recombinant proteins):**<br>Early Secretory Antigen Target, 6 kD (rdESAT-6) + Culture Filtrate Protein,<br>10 kD (rCFP-10)		
**Title:**	An open phase I clinical trial on the safety and the risk of sensitisation by escalating doses and repeated injections of the rdESAT-6 + rCFP-10 skin test reagent following intradermal administration to healthy adults	
**Trial code:**	TESEC-01	
**EudraCT number:**	2008-001489-96	
**Trial phase:**	Phase I (first in man)	
**Investigators:**	Åse Bengård Andersen	
**Investigational centre:**	Rigshospitalet, Denmark	
**Primary objective:**	To assess the safety of three dose levels (0.01, 0.1 and 1.0 µg) of the rdESAT-6 + rCFP-10 skin test reagent when injected to healthy adults	
**Secondary objective:**	To assess the risk of sensitisation of two dose levels (0.01 and 0.1 µg) of the rdESAT-6 + rCFP-10 skin test reagent when injected twice with time intervals of 6 or 12 weeks to healthy adults	
**Primary variable:**	Local and systemic adverse events with onset after the FIRST (0.01, 0.1 and 1.0 µg) or SECOND (0.01 and 0.1 µg) injection.<br>Induration will be regarded as an effect variable and will not be categorized as a local adverse event.<br>Erythema ≥ 20 mm will be regarded as a local adverse reaction.	
**Secondary variable(s):**	1. Sensitisation: The diameter of induration at the second injection site measured transversely to the long axis of the forearm 72 hours after the SECOND skin test injection (0.01 and 0.1 µg). Induration ≥ 6 mm will be regarded as a possible sensitisation reaction<br>2. Sensitisation: QuantiFERON® - TB Gold in tube test results (*in vitro* IFN-γ responses) measured in blood samples taken 28 days after the SECOND skin test injection (0.01 and 0.1 µg). An IFN-γ response of ≥ 0.35 IU/mL will be regarded as a possible sensitisation reaction	

<table>
<tr><td>Name of sponsor:
Statens Serum Institut</td><td rowspan="3"></td><td rowspan="3"></td></tr>
<tr><td>Name of finished product:
rdESAT-6 + rCFP-10 skin test</td></tr>
<tr><td>Names of active ingredient:s (recombinant proteins):
Early Secretory Antigen Target, 6 kD (rdESAT-6) + Culture Filtrate Protein,
10 kD (rCFP-10)</td></tr>
<tr><td>Trial design:</td><td colspan="2">This clinical trial is an open phase I clinical trial investigating the safety and the risk of sensitisation of the rdESAT-6 + rCFP-10 skin test.

The w/w ratio of a mixture of rdESAT-6 / rCFP-10 is 1:1 in all test solutions. For example a dose level of 0.01 µg, refers to a test solution consisting of 0.005 µg rdESAT-6 and 0.005 µg rCFP-10 per 0.1 mL.

Three dose levels will be investigated. The dose levels of 0.01 and 0.1 µg will be injected twice with time intervals of 6 and 12 weeks (Groups A to D). The 1.0 µg dose level will be injected once (Group E). The Mantoux injection technique will be used for all injections in all groups. In total 50 healthy adults will be included into the 5 groups, 10 subjects per group:

Group A: two injections 0.01 µg rdESAT-6 + rCFP-10 (6 weeks interval)
Group B: two injections 0.01 µg rdESAT-6 + rCFP-10 (12 weeks interval)
Group C: two injections 0.1 µg rdESAT-6 + rCFP-10 (6 weeks interval)
Group D: two injections 0.1 µg rdESAT-6 + rCFP-10 (12 weeks interval)
Group E: one injection 1.0 µg rdESAT-6 + rCFP-10

For safety reasons there will be at least 1 hour between the injection of skin test reagent to different subjects.

In groups A to D each healthy female or male will complete in total 6 visits. At Visit 1 (Day 0 to -28), informed consent is obtained, and subsequently, medical examination, laboratory testing and an interview about possible tuberculosis risk factors is performed. Furthermore, BCG vaccination and previous tuberculin skin testing details are recorded. Finally, a blood sample is taken for the QuantiFERON®-TB Gold in tube test. Only subjects that are eligible for inclusion according to the inclusion and exclusion criteria may proceed to the next visit.

At Visit 2 (Day 0) the subjects that are eligible for inclusion are included in the trial and 0.01 ug (Groups A and B) or 0.1 ug (Groups C and D) rdESAT-6 + rCFP-10 skin test is administered in the RIGHT forearm by the Mantoux injection technique.

72 hours after administration of the first skin test (Visit 3), local induration and/or erythema at the injection site is measured and recorded according to the procedures specified in the protocol.

The second skin test is administered at Visit 4 (the time intervals between first and second test administration for groups A to D are shown above). The second dose is administered in the LEFT forearm by the Mantoux injection technique. Just before the second administration a blood sample for the QuantiFERON®-TB Gold in tube test is taken.

72 hours after administration of the second dose (Visit 5), local induration and/or erythema is measured and recorded.

Finally, at the last visit (Visit 6), a medical examination, safety laboratory testing and a blood sample for QuantiFERON®-TB Gold in tube testing is taken.</td></tr>
</table>

**Name of sponsor:**<br>Statens Serum Institut		
**Name of finished product:**<br>rdESAT-6 + rCFP-10 skin test		
**Names of active ingredient:s (recombinant proteins):**<br>Early Secretory Antigen Target, 6 kD (rdESAT-6) + Culture Filtrate Protein,<br>10 kD (rCFP-10)		
**Trial design:**	Diaries are given to the subjects for the recording of adverse events and concomitant medication after first and second skin test administrations. At Visits 2, 3, 4, 5 and 6 adverse events and concomitant medications are assessed and recorded in the CRF by the investigator/study nurse.<br>In group E (see above), where only one injection of skin test (1.0 µg) is given, each healthy female/male will complete in total 4 visits. Visit 1, 2 and 3 will be equal to visits 1, 2, 3 in groups A to D. Visit 4 will be equal to Visit 6 in Groups A to D.<br>Volunteers in the lowest dose groups A and B (0.01 µg rdESAT-6 + rCFP-10) will be included first and tested before proceeding to the next dose level. If no safety concerns are detected within 72 hours after the first injection in the last volunteer of group A, group C and D volunteers (0.1 µg) may be given their first injection. If no safety concerns are detected within 72 hours after the first injection in the last volunteer of group C volunteers in group E (1.0 µg) may be given their first injection.	
**Trial population:**	Healthy, non-black, female/male adults (≥ 18 years of age) who are QuantiFERON® TB Gold in tube negative at inclusion	
**Planned number of subjects:**	50 subjects allocated to 5 groups of each 10 subjects	
**Inclusion criteria:**	**The subject:**<br>1. Has signed an informed consent<br>2. Is willing and likely to be able to comply with the trial procedures<br>3. Is female/male non-black adult ≥ 18 years and ≤ 55 years of age<br>4. Is healthy according to a medical examination, medical history and laboratory investigations at inclusion<br>5. Is prepared to grant authorized persons access to their medical records	

**Name of sponsor:**<br>Statens Serum Institut		
**Name of finished product:**<br>rdESAT-6 + rCFP-10 skin test		
**Names of active ingredient:s (recombinant proteins):**<br>Early Secretory Antigen Target, 6 kD (rdESAT-6) + Culture Filtrate Protein, 10 kD (rCFP-10)		
**Exclusion criteria:**	**The subject:**<br>1. Has a history of tuberculosis or has had a known contact to a person with active tuberculosis<br>2. Has a positive QuantiFERON®-TB Gold In Tube test result at inclusion<br>3. Laboratory parameters outside of normal range judged by principal investigator to be clinically significant and/or abnormal glucose or protein levels in a urine sample at inclusion<br>4. Has been in treatment with a product which is likely to modify the immune response within 3 months prior to the day of inclusion (e.g. immunoglobulin, systemic corticosteroids, methotrexate, azathioprine, cyclosporine, infliximab or blood products)<br>5. Has been vaccinated with a live vaccine within 6 months prior to the day of inclusion (e.g. MMR, yellow fever, or oral typhoid vaccines)<br>6. Has a known congenital or acquired immune defiency<br>7. Has a disease affecting the lymphoid organs (e.g Hodgkin's disease, lymphoma, leukaemia, sarcoidosis)<br>8. Is known to be infected with HIV, HBV or HCV<br>9. Has a severe ongoing viral or bacterial infection that might affect the cell mediated immune response at inclusion<br>10. Has a C-reactive protein (CRP) level > 50 mg/L<br>11. Has severe scarring, burn, rash, eczema, psoriasis, or any other skin disease at or near the injection site(s)<br>12. Has a condition in which repeated blood drawings pose more than minimal risk for the volunteer, such as haemophilia, other coagulation disorders, or significantly impaired venous access<br>13. Is actively participating in another clinical trial or has participated in previous clinical trials investigating the rdESAT-6 skin test reagent<br>14. Is pregnant according to urine pregnancy test at inclusion<br>15. Is a female not willing to use contraceptives or is breastfeeding<br>16. Has a condition which in the opinion of the investigator is not suitable for participation in the study	
**Investigational diagnostic skin test::**	**Investigational product rdESAT-6 + rCFP-10**<br>rdESAT-6 .... 0.05/0.5/5.0 µg<br>rCFP-10 .... 0.05/0.5/5.0 µg<br>Disodium hydrogen phosphate dihydrate .... 1.4 mg<br>Potassium dihydrogen phosphate .... 0.2 mg<br>Potassium chloride .... 0.2 mg<br>Sodium chloride .... 8.0 mg<br>Polysorbate 20 .... 0.1 µL<br>Water for injections .... up to 1 mL	
**Doses and route of administration:**	0.1 mL of the investigational diagnostic test is administered intradermally by the Mantoux technique in the dorsal aspect of the RIGHT or LEFT forearm. A 1 mL syringe fitted with a short bevelled needle (insulin needle) is used for the injection of the test.	
**Statistical methods:**	Descriptive methods.	

2. Table of Contents

1. Synopsis 1
2. Table of Contents 5
3. List of Abbreviations and Definitions 7
4. Signature Page 9
5. Relevant Addresses 10
6. Introduction 12
7. Trial objectives 14
7.1. Primary objective 14
7.2. Secondary objective 14
7.3. Primary variable 14
7.4. Secondary variables 15
8. Investigational Plan 15
8.1. Overall design 15
8.2. Study population 17
8.2.1. Recruitment 17
8.3. Inclusion criteria 17
8.4. Exclusion criteria 18
8.5. Predetermined reasons for discontinuation 19
8.6. Handling of withdrawn volunteers and drop-outs 19
9. Investigational Products 19
9.1. Treatments administered 19
9.1.1. Composition 20
9.1.2. Doses and administration 20
9.1.3. Packaging and labelling 22
9.1.4. Storage information 22
9.1.5. Samples of labels for test product 22
9.1.6. Transport of study products 22
9.1.7. Randomisation procedure 22
9.1.8. Treatment compliance 23
9.1.9. Drug accountability 23
9.1.10. Precautions and overdosing 23
9.1.11. Concomitant medication 23
9.1.12. Investigational events 24
9.1.13. Allocation of volunteers to study groups 25
9.1.14. Assessment of safety 25
9.1.15. Assessment of sensitisation 26
9.1.16. Collection and handling of blood samples 27
9.1.17. Laboratory assays 28
9.1.18. Time schedule 28

10. Ethical Aspects 28
10.1. Payment of Volunteers 30
10.2. Data Safety Monitoring Plan 30
11. Adverse Events 31
11.1. Definitions and terms 31
11.2. Standard reporting of adverse events 33
11.3. Expedited reporting of adverse events 34
12. Data Management and Statistical Analysis 34
12.1. General considerations 34
12.2. Data management 34
12.3. Clean file procedures 35
12.4. Analysis populations 35
12.5. Statistical methods 35
12.6. Sample size determinations 35
13. Good clinical practice considerations 36
13.1. Declaration of Helsinki 37
13.2. Volunteer information and informed consent 37
13.3. Independant Ethics Committee submission and approval 37
13.4. Regulatory health authority submission and approval 37
13.5. Volunteer data protection 38
13.6. Investigator's responsibilities 38
13.7. Curricula vitae and log(s) of staff 38
13.8. Indemnity statement 38
13.9. Training 39
13.10. Monitoring 39
13.11. Audit and inspection 39
13.12. Definition of source data 39
13.13. Archiving of essential documents 40
14. Agreement and financial settlement 40
15. Insurance 40
16. Confidentiality and disclosure 41
17. Changes to the protocol 41
18. References 41

Appendices

Appendix 1: CIOMS Form

Appendix 2: Declaration of Helsinki

Appendix 3: Participant Information Sheet

Appendix 4: Informed Consent Form

Appendix 5: Indemnity Statement

Appendix 6: Insurance Certificate

Appendix 7: Mantoux testing technique

Appendix 8: "Før du beslutter dig"

3. List of Abbreviations and Definitions

ADR	Adverse Drug Reaction
AE	Adverse Event
ALP	Alkaline Phosphatase
ALT	Alanine aminotransferase
AR	Adverse Reaction
AST	Aspartate aminotransferase
BCG	Bacille Calmette-Guérin
CFP-10	Culture Filtrate Protein -10CI
CI	Confidence Interval
CRF	Case Record Form
DTH	Delayed Type Hypersensitivity
DSMB	Data safety monitoring board
DSMP	Data safety monitoring plan
EC	Ethics Committee
ESAT-6	Early Secretory Antigenic Target
FPFV	First Patient First Visit
GCP	Good clinical practice
GMP	Good manufacturing practice
HBV	Hepatitis B virus
HBC	Hepatitis C virus
HIV	Human Immunodeficiency Virus
IB	Investigator's Brochure
IMPD	Investigational medicinal product dossier
IF	Investigator's file
IFN-γ	Interferon gamma
LPLV	Last Patient Last Visit
NCS	Not clinically significant
PPD RT 23	Purified Protein Derivative Rinsed Tuberculin (batch) 23
QFT	QuantiFERON®-TB Gold In Tube test
rCFP-10	Recombinant 10kDa Culture Filtrate Protein
rdESAT-6	Recombinant dimer of 6 kDa Early Secreted Antigen Target
RBC	Red blood cell count
SAE	Serious Adverse Event
Sensitivity	The probability that a test result is positive given the subject has the disease [30]
SPC	Summary of Product Characteristics
Specificity	The probability that a test result is negative given the subject does not have the disease [30]
SSI	Statens Serum Institut, Denmark
SSAR	Serious Suspected Adverse Reaction
SAR	Suspected adverse (drug) reaction

SUSAR	Suspected Unexpected Serious Adverse (drug) Reaction
TB	Tuberculosis
TMF	Trial master file
TST	Tuberculin Skin Test
WBC	White blood cell count

4. Signature Page

Study director and monitor, SSI:	**Pernille Nyholm Tingskov**	______________________ (date & signature)
Co - study director, approval of monitoring, SSI:	**Birgit Thierry-Carstensen**	______________________ (date & signature)
Project manager, SSI:	**Henrik Aggerbeck**	______________________ (date & signature)
Principal investigator:	**Åse Bengård Andersen**	______________________ (date & signature)
Statistician, SSI:	**Anders Mørup Jensen**	______________________ (date & signature)
Responsible Medical Advisor, SSI	**Trine R. Nielsen**	______________________ (date & signature)
Head of Department of vaccine development, SSI:	**Ingrid Kromann**	______________________ (date & signature)

5. Relevant Addresses

**Principal Investigator:**	Åse Bengård Andersen<br>Department of Infectious Diseases<br>M 51-3-2<br>Rigshospitalet<br>Blegdamsvej 9<br>2100 København Ø	Tel: +45 35458555<br>Fax: +45 35456648<br>e-mail: bengaard@rh.dk
**Investigator:**	Name unknown	Tel:<br>Fax:<br>e-mail:
**Study Director & Monitor:**	Pernille Tingskov<br>Statens Serum Institute<br>Department of<br>Vaccine Development<br>5 Artillerivej<br>DK-2300 Copenhagen S	Tel: +45 3268 3416<br>Fax: +45 3268 3872<br>e-mail: pnt@ssi.dk
**Co-study director, approval of monitoring:**	Birgit Thierry-Carstensen<br>Statens Serum Institute<br>Department of<br>Vaccine Development<br>5 Artillerivej<br>DK-2300 Copenhagen S	Tel: +45 3268 3598<br>Fax: +45 3268 3872<br>e-mail: btc@ssi.dk
**Project manager:**	Henrik Aggerbeck<br>Statens Serum Institute<br>Department of<br>Vaccine Development<br>5 Artillerivej<br>DK-2300 Copenhagen S	Tel: +45 3268 3486<br>Fax: +45 3268 3872<br>e-mail: hea@ssi.dk
**Responsible Medical Advisor:**	Trine R. Nielsen<br>Statens Serum Institute<br>Department of Regulatory and<br>Medical Affairs<br>5 Artillerivej<br>DK-2300 Copenhagen S	Tel: +45 3268 3436<br>Fax: +45 3268 3973<br>e-mail: trn@ssi.dk
**Head of Department of vaccine development:**	Ingrid Kromann<br>Statens Serum Institute<br>Department of<br>Vaccine Development<br>5 Artillerivej<br>DK-2300 Copenhagen S	Tel: +45 3268 8252<br>Fax: +45 3268 3972<br>e-mail: ikr@ssi.dk
**Statistician:**	Anders Mørup Jensen<br>Statens Serum Institute<br>Biostatistics Unit<br>5 Artillerivej<br>DK-2300 Copenhagen S	Tel: +45 3268 3284<br>Fax: +45 3268 3886<br>e-mail: amo@ssi.dk

**Head of the Data Safety Monitoring Board:**	Dr. Sandra M. Arend<br>Department of Infectious Diseases<br>C5P-39, LUMC, PO Box 9600, 2300 RC Leiden,<br>The Netherlands	Tel: +31 71 5262620<br>Fax: +31 71 5266758<br>e-mail: S.M.Arend@lumc.nl
**Member of the Data Safety Monitoring Board:**	Dr.Thomas Benfield<br>Department of Infectious Diseases<br>Hvidovre Hospital (1330 580)<br>Kettegårds Allé 30, 144/112<br>2650 Hvidovre<br>Denmark	Tel: +45 36322932<br>Fax: +45 36323405<br>e-mail: tlb@dadlnet.dk
**Member of the Data Safety Monitoring Board:**	Dr. PhD Pernille Ravn<br>Medical Department Q<br>Herlev Hospital<br>Herlev Ringvej 75<br>2730 Herlev<br>Denmark	Tel: +45 44884488<br>e-mail: pravn@dadlnet.dk

Emergency telephone numbers and e-mail address:

**Study Director & monitor, SSI:**	Pernille Nyholm Tingskov	Office: +45 3268 3416<br>Mobile: +45 5176 1222
**Co–study director, SSI:**	Birgit Thierry-Carstensen	Office: +45 3268 3598<br>Mobile: +45 2683 8437<br>Private: +45 3543 4439
**Medically Responsible, SSI:**	Trine R. Nielsen	Office: +45 3268 3436<br>Mobile: +45 2680 3003
**Serious Adverse Event Notification to SSI:**		Fax: +45 3268 3973<br>Mobile: +45 2567 0914<br>e-mail : clin.trial@ssi.dk

6. Introduction

Tuberculosis continues to be a major cause of morbidity and mortality throughout the world. The disease is caused by infection with *Mycobacterium tuberculosis* (*M. tuberculosis)* and remains one of the most important fatal infections of human beings, with 9 million new cases every year and an estimated 2 million deaths [1].

To control the disease fast and accurate diagnosis is very important; however in many clinical situations this is not possible as the existing diagnostic methods have serious limitations [13]. The tuberculin skin test (TST) with Purified Protein Derivative (PPD) has been used world-wide for almost 100 years to support the diagnosis of tuberculosis (TB) as well as for screening in national TB programs and epidemiological studies [2, 3]. The drawback of PPD is the fact that its protein components are known to be shared by many non-tuberculous/environmental mycobacterium families as well as by the BCG vaccine strains. This fact significantly decreases the specificity of the Tuberculin Skin Test (TST), since individuals exposed to non-tuberculous mycobacteria or vaccinated with BCG respond immunologically to PPD as well as those who are infected with the tuberculous families (*M. tuberculosis*, *M. bovis,* or *M. africanum*) [4].

Recently it has been discovered that the tuberculous bacteria contains a number of specific proteins/antigens that are not present in BCG or most environmental mycobacteria. The ESAT-6 (6 kD Early Secreted Antigen Target) protein and CFP-10 (10 kDa culture filtrate protein-10) were identified from a *M. tuberculosis* culture filtrate [5, 28]. Both antigens, ESAT-6 and CFP-10 are expressed by the tuberculous mycobacterium families (*M. tuberculosis, M. bovis, and M. africanum*) but not by any of the BCG-strains and only very few of the atypical mycobacteria express the proteins (e.g., *M. kansasii, M. marinum, M. szulgai*) [4, 28].

Based on this knowledge new in-vitro diagnostic tests have been developed in the form of T-cell based interferon γ-assays. These assays use *Mycobacterium tuberculosis* - specific antigens (e.g. ESAT-6, CFP-10) and show that T-cells of individuals infected with *M. tuberculosis* produce interferon-γ when they are exposed to these antigens [14]. The tests show very promising abilities in detecting latent infection with *M. tuberculosis* and were shown to be able to discriminate between patients infected by *M. tuberculosis* and BCG vaccinated individuals [6, 7, 8, 9, 28].

A new skin test using the rdESAT-6 + rCFP-10 antigens has been developed by SSI, Denmark and has shown to induce local delayed type hypersensitivity (DTH) skin reactions when injected intradermally to guinea pigs infected with *M. tuberculosis* [28, 36, 37].

In 2005/2006 the rdESAT-6 reagent (alone) synthesised in *Lactococcus lactis,* was administered to humans for the first time in a dose escalating phase 1 clinical trial conducted at Leiden University Medical College, the Netherlands. The rdESAT-6 was administered at 4 different dose levels (0.01 µg, 0.1 µg, 1 µg and 10 µg) in a group of healthy volunteers and later at 3 different dose levels (0.01 µg, 0.1 µg and 1 µg) in a group of previously treated TB-patients. The aim of this first in man phase 1 trial was to investigate the safety and the diagnostic potential of the new skin test reagent rdESAT-6 and to compare it with 2 T.U. PPD. No significant safety issues were identified. The rdESAT-6 skin test induced delayed type hypersensitivity reactions in treated TB

patients at all tested dose levels. Furthermore reactions of similar size in the groups injected with 0.1 µg rdESAT-6 and 2 T.U. PPD (in separate arms) were observed. Based on these observations a dose level of 0.1 µg rdESAT-6 was selected for the next phase 1 clinical trial. [29].

Results from non clinical pharmacology studies in guinea pigs have demonstrated a risk of sensitisation upon repeated injections of rdESAT-6 + rCFP-10 visualized by a local induration and erythema at the injection site. To address this issue a phase 1b clinical trial was conducted at Rigshospitalet , Copenhagen in 2007 to assess the risk of sensitisation upon repeated injections of a fixed dose level of 0.1 µg rdESAT-6 with time intervals of 28, 56 or 112 days, respectively.

In this phase 1b clinical trial with the rdESAT-6 reagent (alone) it was documented that repeated injections of the rdESAT-6 can be given safely, but that positive reactions may occur due to sensitisation. However, the risk may be diminished by a proper time span between succeeding testing [31].

In March 2007, SSI decided to include rCFP-10 to increase the diagnostic performance of the skin test reagent reflected by an improved sensitivity without jeopardizing the specificity of the skin test reagent [28]. Non clinical pharmacology trials at SSI demonstrated in guinea pigs that rCFP-10 mixed with rdESAT-6 in the ratio 1:1 was optimal.

Due to the above change in the development plan the present clinical trial has been designed to investigate the safety and the risk of sensitisation of the improved skin test reagent in healthy adult volunteers. It will be the first in man clinical trial using a mixture of rdESAT-6 + rCFP-10. Three dose levels of 0.01, 0.1 and 1.0 µg rdESAT-6 + rCFP-10 mixed in the ratio 1:1 (w/w) will be investigated. Furthermore the possible risk of sensitisation of the rdESAT-6 + rCFP-10 skin test will be investigated by giving two test doses of 0.01 and 0.1 µg skin test reagent with time intervals of 6 or 12 weeks, respectively.

Reece et al [33] recently suggested that CFP-10 may trigger "Tuberculin shock". He found that 5/10 guinea pigs died 6-36 hours after the skin testing with CFP-10, if the test was done 6 weeks after IV infection. No deaths were observed if the skin testing was done after 4 weeks or earlier. A more likely explanation is that the testing and the deaths were coincidental as the guinea pigs after 6 weeks of infection were at a late stage of disease. In a repeat study No. F1137 [40] done at SSI with an infection period of 6 weeks before testing, 3/30 (10%) animals died or were about to die from tuberculosis even before skin testing. The same number of animals died after skin testing with rCFP-10 or PPD. It is likely that an immunological reagent at this time of disease will be able to induce a shock-like syndrome in the animals.

Non clinical toxicity studies performed in 2008 concluded that repeated subcutaneous injections of 10 µg rdESAT-6 +rCFP-10 were safe in rats.

Based on the nonclinical pharmacology and toxicity studies and two phase I clinical trials with rdESAT-6 (alone), it is expected that administration of rdESAT-6 + rCFP-10 skin test by the Mantoux injection technique in doses between 0.01 and 1.0 µg only will expose the volunteers to MINIMAL risks, such as local injection site reactions.

Injection of the rdESAT-6 + rCFP-10 skin test reagent is intended to identify latent tuberculosis infection by the formation of a local erythema and induration at the injection site 48 h - 96 h after the injection (positive reaction). No reaction is expected to occur in uninfected persons after a single injection. In the present study a skin reaction after the first injection will be regarded as a sign of latent infection or an adverse reaction. We will investigate if a positive skin reaction occurs after a second injection of rdESAT-6 + rCFP-10 given to healthy individuals at an interval of 6 or 12 weeks. In case such a response appears it may be regarded as a sensitisation reaction induced by the preceding injection. Such a false positive skin reaction appears similar to a positive skin reaction in a TB infected person. To the individual the response does not represent a safety issue, but it is an undesired property by the reagent.

50 male/female healthy adult volunteers will be allocated to 5 groups (A, B, C, D and E) of 10 participants. The dose levels of 0.01 and 0.1 µg rdESAT-6 + rCFP-10 will be injected twice with time intervals of 6 and 12 weeks, respectively (Group A to D). The 1.0 µg dose level will only be injected once (Group E). Details of the immunological status of each volunteer (BCG vaccination, suspected mycobacterial exposure etc.) will be recorded at study entry.

7. Trial objectives

7.1. Primary objective

To assess the safety of three dose levels (0.01, 0.1 and 1.0 µg) of the rdESAT-6 + rCFP-10 skin test reagent when injected to healthy adults.

7.2. Secondary objective

To assess the risk of sensitisation of two dose levels (0.01 and 0.1 µg) of the rdESAT-6 + rCFP-10 skin test reagent when injected twice with time intervals of 6 or 12 weeks to healthy adults.

7.3. Primary variable

Local and systemic adverse events with onset after the FIRST (0.01, 0.1 and 1.0 µg) or SECOND (0.01 and 0.1 µg) injection.

Induration will be regarded as an effect variable and will not be categorized as a local adverse event.

Erythema ≥ 20 mm will be regarded as a local adverse reaction.

7.4. Secondary variables

- Sensitisation: The diameter of induration at the second injection site measured transversely to the long axis of the forearm 72 hours after the SECOND skin test injection (0.01 and 0.1 µg). Induration ≥ 6 mm will be regarded as a possible sensitisation reaction.

- Sensitisation: QuantiFERON® - TB Gold in tube test results (*in-vitro* IFN-γ responses) measured in blood samples taken 28 days after the SECOND skin test injection (0.01 and 0.1 µg). An IFN-γ response of ≥ 0.35 IU/mL will be regarded as a possible sensitisation reaction.

8. Investigational Plan

This phase I clinical trial has been planned in accordance with 'Note for guidance on good clinical practice', CPMP/ICH/135/95, ICH Topic E 6 [18]. It is an open phase 1 clinical trial to investigate the safety and the risk of sensitisation by escalating doses and repeated injections of the rdESAT-6 + rCFP-10 skin test reagent following intradermal administration to healthy adults. Three dose levels of the rdESAT-6 + rCFP-10 reagent will be investigated (0.01, 0.1 and 1.0 µg). The dose levels of 0.01 and 0.1 µg will be injected twice with time intervals of 6 or 12 weeks.

8.1. Overall design

The trial is a single-centre phase 1 clinical trial and will be conducted at the Department of Infectious Diseases at Rigshospitalet, Blegdamsvej 9, 2100 København Ø, Denmark, under the responsibility of principal investigator: Åse Bengård Andersen.

The primary objective is to assess the safety of the new *in-vivo* rdESAT-6 + rCFP-10 reagent with three different dose levels of 0.01, 0.1 and 1.0 µg.

The potential sensitisation risk of rdESAT-6 + rCFP-10 will primarily be evaluated by measuring the diameter of induration at the injection site 72 hours after the SECOND skin test injection (i.e reading the diameter in millimetres). An induration of ≥ 6 mm will be regarded as a possible sensitisation reaction.

In addition, the *in vitro* interferon-γ response results from the QuantiFERON® TB Gold in tube tests taken at the screening visit, prior to administration of the second rdESAT-6 + rCFP-10 dose and 28 days after the second rdESAT-6 + rCFP-10 dose will be compared (i.e. secondary sensitisation risk variable). An IFN-γ response of ≥ 0.35 IU/mL 28 days after the SECOND rdESAT-6 + rCFP-10 injection will be regarded as a possible sensitisation reaction.

50 healthy non-black male and/or female adult volunteers ≥ 18 and ≤ 55 years of age will be included in the trial.

Three dose levels will be investigated. The dose levels of 0.01 and 0.1 µg will be injected twice with a timeinterval of 6 and 12 weeks, respectively (Groups A, B, C and

D). The final dose level of 1.0 µg will only be injected once (Group E). The Mantoux injection technique will be used for all injections in all groups.

For safety reasons there will be at least 1 hour between the injections of the rdESAT-6 + rCFP-10 skin test reagent to different volunteers.

Volunteers in the lowest dose groups A and B (0.01 µg rdESAT-6 + rCFP-10) will be included first and tested before proceeding to the next dose level. If no safety concerns are detected within 72 hours after the first injection in the last volunteer of group A, group C and D volunteers (0.1 µg) may be given their first injection. If no safety concerns are detected within 72 hours after the first injection in the last volunteer of group C volunteers in group E (1.0 µg) may be given their first injection.

The first dose of the rdESAT-6 + rCFP-10 reagent (0.01 or 0.1 µg) will be injected on day 0 in the RIGHT forearm of the volunteers in Groups A, B, C and D. A follow-up visit will take place 72 hours after the first dose.

The second dose will be administered on day 42 of the LEFT forearm of the volunteers in Group A (0.01 µg) and Group C (0.1 µg) or on day 84 in Group B (0.01 µg) and Group D (0.1 µg), with follow-up visits after 72 hours and after 28 days, respectively.

Volunteers allocated to Group E will only be injected with one rdESAT-6 + rCFP-10 injection with the high dose level of 1.0 µg in the RIGHT forearm. Follow up visits will take place 72 hours and 28 days after the FIRST and only rdESAT-6 + rCFP-10 injection.

For an overview of the injection schedule for all groups (A, B, C, D and E) please refer to following table:

**Dose**	**Group**	**Day 0**	**Day 42**	**Day 84**
0.01 µg rdESAT-6 + rCFP-10	A	×	×	
	B	×		×
0.1 µg rdESAT-6 + rCFP-10	C	×	×	
	D	×		×
1.0 µg rdESAT-6 + rCFP-10	E	×		

The volunteers are monitored closely for the occurrence of immediate adverse reactions during the first hour after ALL injections in ALL groups.

Each trial volunteer will be followed 28 days after the last skin test injection.

Volunteers allocated to Groups A, B, C and D are expected to complete a total of 6 trial visits, as follows:

Visit 1 Screening Visit
Visit 2 *Inclusion Visit and Injection of the FIRST skin test. (Day 0)*
Visit 3 *72-hour Assessment Visit after the FIRST skin test*
Visit 4 *Injection of the SECOND skin test (Days 42 or 84, respectively)*
Visit 5 *72-hour Assessment Visit after the SECOND skin test*
Visit 6 *Day 28 Assessment Visit after the SECOND skin test*

Volunteers allocated to Group E are expected to complete a total of 4 Visits as follows:

Visit 1 Screening Visit
Visit 2 *Inclusion Visit and Injection of the FIRST skin test. (Day 0)*
Visit 3 *72-hour Assessment Visit after the FIRST skin test*
Visit 4 *Day 28 Assessment Visit after the FIRST skin test*

For practical reasons Visit 2 should always take place on a Monday, Tuesday or Friday. For more details on the visits see Section 9.1.12.

Volunteers with a positive sensitisation reaction or a positive QuantiFERON test result will be asked to return for a final check 6 months after the last visit in the trial to exclude an infection with tuberculosis.

8.2. Study population

Five groups of each 10 male or female QFT negative healthy adult volunteers will be included. Pregnant and/or breastfeeding female volunteers will be excluded from participation in the trial.

To obtain the optimal conditions for an accurate visual evaluation of the size of the skin test reactions (induration and/or redness) volunteers with black skin will not be included.

8.2.1. Recruitment

Healthy adults will mainly be recruited from the University of Copenhagen. An advertisement approved by the Ethics Committee will if needed be published in a national newspaper for further recruitment.

8.3. Inclusion criteria

The volunteer:

1. Has signed an informed consent
2. Is willing and likely to be able to comply with the trial procedures
3. Is female/male non-black adult ≥ 18 years and ≤ 55 years of age

4. Is healthy according to a medical examination, medical history and laboratory investigations at inclusion
5. Is prepared to grant authorized persons access to their medical records

8.4. Exclusion criteria

The volunteer:

1. Has a history of tuberculosis or has had a known contact to a person with active tuberculosis
2. Has a positive QuantiFERON®-TB Gold In Tube test result at inclusion
3. Laboratory parameters outside of normal range judged by principal investigator to be clinically significant and/or abnormal glucose or protein levels in a urine sample at inclusion
4. Has been in treatment with a product which is likely to modify the immune response within 3 months prior to the day of inclusion (e.g. immunoglobulin, systemic corticosteroids, methotrexate, azathioprine, cyclosporine, infliximab or blood products)
5. Has been vaccinated with a live vaccine within 6 months prior to the day of inclusion (e.g. MMR, yellow fever, or oral typhoid vaccines)
6. Has a known congenital or acquired immune defiency
7. Has a disease affecting the lymphoid organs (e.g Hodgkin's disease, lymphoma, leukaemia, sarcoidosis)
8. Is known to be infected with HIV, HBV or HCV
9. Has a severe ongoing viral or bacterial infection that might affect the cell mediated immune response at inclusion
10. Has a C-reactive protein (CRP) level > 50 mg/L
11. Has severe scarring, burn, rash, eczema, psoriasis, or any other skin disease at or near the injection site(s)
12. Has a condition in which repeated blood drawings pose more than minimal risk for the volunteer, such as haemophilia, other coagulation disorders, or significantly impaired venous access
13. Is actively participating in another clinical trial or has participated in previous clinical trials investigating the rdESAT-6 skin test reagent
14. Is pregnant according to urine pregnancy test at inclusion
15. Is a female not willing to use contraceptives or is breastfeeding
16. Has a condition which in the opinion of the investigator is not suitable for participation in the study

8.5. Predetermined reasons for discontinuation

Trial volunteers are free to withdraw from the clinical trial whenever they desire. Volunteers withdrawn from or discontinuing the trial after the FIRST injection should (if possible) for safety concerns stay in the trial for the follow up visits but will **not** receive the SECOND injection. These volunteers will be replaced by new volunteers. If the volunteer is withdrawn or discontinues the trial after the SECOND injection he or she will not be replaced.

No reaction is expected to occur in uninfected persons after a single injection. In the present study a skin reaction after one injection will be regarded as sign of latent infection or an adverse reaction and the volunteer should (if possible) for safety concerns stay in the trial for the follow-up visits but must NOT receive the SECOND vaccination. The volunteer should in this case be replaced by a new volunteer.

If, for any reason, a volunteer wishes to discontinue her/his participation in the trial, or if, according to principal investigator's judgement, she/he must be withdrawn from the trial, the date and reason for the withdrawal must be recorded in the CRF.

The trial can be terminated at any time if the sponsor, the principal investigator or the independent data safety monitoring board decides that the trial poses an unacceptable threat to the volunteers.

8.6. Handling of withdrawn volunteers and drop-outs

For withdrawn volunteers and for volunteers who drop out before the planned last visit, a Termination Visit will be performed. At the Termination Visit, a full medical examination will be performed, blood and urine samples will be collected, and adverse events, if any, will be assessed and recorded. If an individual has special needs, proper medical action will be taken. Such action will be at the discretion of the investigator.

All data will be included in the safety and efficacy analysis of the results, including data from withdrawn volunteers or volunteers who dropped out of the trial before the planned last visit.

9. Investigational Products

9.1. Treatments administered

The rdESAT-6 + rCFP-10, is manufactured at SSI in Denmark according to Good Manufacturing Practice (GMP) standards.

The w/w ratio of a mixture of rdESAT-6 and rCFP-10 is 1:1 in all test solutions. For example a dose level of 0.01 µg, refers to a test solution consisting of 0.005 µg rdESAT-6 and 0.005 µg rCFP per 0.1 mL.

The rdESAT-6 + rCFP-10 reagent is formulated as a solution for injection in the following strengths:

0.01 µg rdESAT-6 + rCFP-10 / 0.1 mL
0.1 µg rdESAT-6 + rCFP-10 / 0.1 mL
1.0 µg rdESAT-6 + rCFP-10 / 0.1 mL

rdESAT-6 + rCFP-10 is dissolved in phosphate buffered saline (PBS) containing 0.01% Polysorbate 20 and with a pH adjusted between 6.5 and 7.5. It appears as a clear, colourless solution.

The test solution is filled into 3 mL glass vials with perforable rubber stoppers and aluminium caps with white, red and blue plastic 'flip-off' tops indicating the following concentrations 0.01, 0.1 and 1.0 µg respectively. Each vial is intended for administration of 0.1 mL to one trial volunteer.

9.1.1. Composition

Investigational products – rdESAT-6 + rCFP-10:

rdESAT-6 ....0.05/0.5/5.0 µg
rCFP-10 ....0.05/0.5/5.0 µg
Disodium hydrogen phosphate dihydrate .... 1.4 mg
Potassium dihydrogen phosphate .... 0.2 mg
Potassium chloride.. 0.2 mg
Sodium chloride.. 8.0 mg
Polysorbate 20.. 0.1 µL
Water for injections ....up to 1 mL

9.1.2. Doses and administration

The rdESAT-6 + rCFP-10 skin test is administered by the Mantoux technique by staff with training and experience in the Mantoux testing technique.

The test product should preferably be taken out of the refrigerator half an hour before each injection is performed.

Before the administration the "used by" date must be checked.

The test may only be administered if it appears as a clear and colourless solution.

Disinfection of the injection site before the injection is not necessary. If the injection site is disinfected anyway, it must be completely dry before the injection of the test product.

Use a sterile 1.0 mL disposable syringe fitted with a large-size needle to aspirate the test fluid from the vial. Replace the needle with a short-bevelled needle 26 gauge and administer 0.1 mL of the test solution by the Mantoux technique as specified by SSI (see appendix 7).

Draw up slightly more than 0.1 mL, remove air bubbles and adjust the volume to exactly 0.1 mL.

Stretch the skin slightly and hold the needle almost parallel to the skin with the bevelled side upwards. The needle should be inserted about 2 mm into the middle third part of the dorsal side of the RIGHT forearm (for the FIRST test dose for volunteers in Groups A, B, C, D and E) and into the middle third part of the dorsal side of the LEFT forearm (for the SECOND test dose for volunteers in Groups A, B, C and D). The needle should be visible through the epidermis before SLOWLY inserting 0.1 mL of the test solution intradermally.

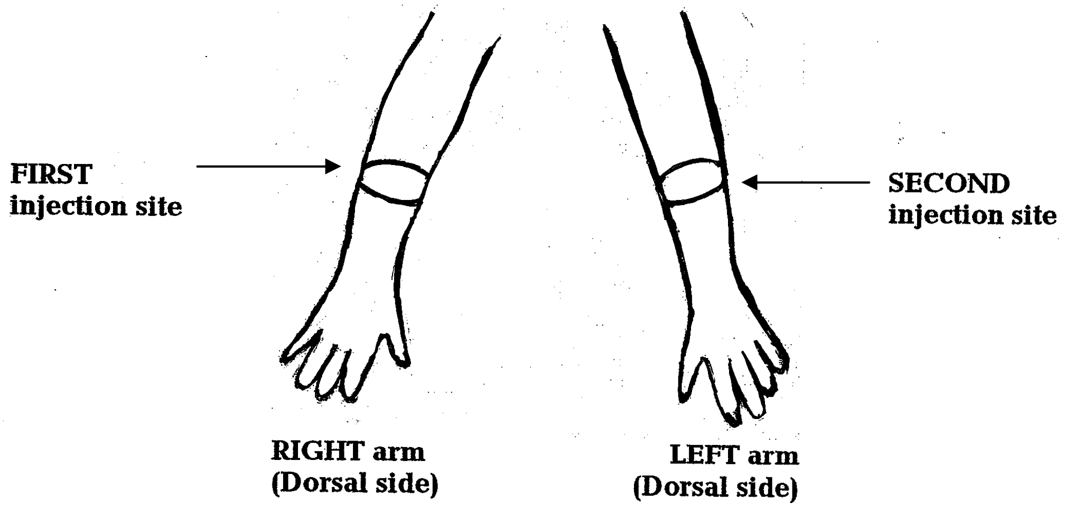

The appearance of a small papule of 8-10 mm in diameter after the injection indicates correct injection technique. The papule disappears after approximately 10 minutes.

If the injection fails, i.e. does not result in a papule (as described above), this must be recorded in the CRF together with the reason. A failed injection must under no circumstances be repeated in the same volunteer. In case the FIRST injection fails in a given volunteer, he/she should (if possible) for safety concerns stay in the trial for the follow-up visits but must NOT receive the SECOND vaccination. The volunteer should in this case be replaced by a new volunteer. If the SECOND vaccination fails, the given volunteer should for safety reasons continue in the trial but should not be replaced by a new volunteer.

No reaction is expected to occur in uninfected persons after a single injection. In the present study a skin reaction after one injection will be regarded as sign of latent infection or an adverse reaction and the volunteer should (if possible) for safety concerns stay in the trial for the follow-up visits but must NOT receive the SECOND vaccination. The volunteer should in this case be replaced by a new volunteer.

The syringe and the needle must be discarded as a single unit after injection in a labelled, puncture-proof container. The vial must be kept together with the cardboard box until drug accountability documents have been completed and approved by the monitor. This will normally take place at the termination monitoring visit.

9.1.3. Packaging and labelling

The packaging and labelling is performed at SSI according to Good Manufacturing Practice (GMP). The vials will be packed in boxes. Each box will contain 10 vials.

9.1.4. Storage information

The shelf-life of the rdESAT-6 + rCFP-10 skin test is 6 months if stored in a refrigerator at + 2ºC to + 8ºC. Stability studies are ongoing at SSI and if the product shows to be stable an extension of the shelf-life may occur during the study.

To avoid exposure to light the vials should be kept in the cardboard boxes until administered. The "used by" date is printed on the vials. The test products are stored in a locked fridge at the Department of Infectious Diseases at Rigshospitalet. The refrigerator where the test products are stored is equipped with a temperature surveillance system and an alarm system. Staff at the Department of Engineering at Rigshospitalet are responsible for the monitoring of storage conditions. Principal investigator Åse Bengård Andersen is responsible for the dispensing of the trial product. The dispensed test solutions will be recorded in a dispensing log.

In case the refrigerator, where the test items are stored, breaks down, the test items should be moved to another refrigerator as soon as possible. The reason and time of when the test items are moved to another location must be registered in the temperature log of the test items.

In case of significant storage condition deviations, as judged by the principal investigator, the items subject to the deviations may not be used and the SSI clinical trial monitor should be contacted as soon as possible for advice.

The SSI clinical monitor will seek information from relevant parties at SSI, in respect to decide whether the test items, which were subject to the deviations, should be destructed or whether they can still be used in the trial.

9.1.5. Samples of labels for test product

Examples of the labels for the cardboard boxes and the vials will be provided as a separate document when submitting the protocol to the Competent Authorities.

9.1.6. Transport of study products

The test product will be transported by car under controlled temperature conditions (2-8 °C). Transport, dispatch and receipt procedures will be documented.

9.1.7. Randomisation procedure

By nature the trial has to be open, but it could in principle be randomised. Randomisation would, however, require that all volunteers who were found eligible at the inclusion visit would have to accept to follow any of the 5 different study schedules before the randomisation took place. This would make it difficult to recruit volunteers for the trial, so it was decided to run the trial without randomisation.

9.1.8. Treatment compliance

As the test products are injected by study staff during the study visits under controlled conditions, treatment compliance procedures are not relevant in the present clinical trial.

9.1.9. Drug accountability

All dispensed test products will be recorded in a dispensing log. All used and unused vials must be kept and returned to SSI to be accounted for at the end of the trial. Procedures in relation to the dispensing and the accountability of the test products will be the responsibility of the principal investigator Åse Bengård Andersen. The trial monitor from SSI will check the records.

9.1.10. Precautions and overdosing

Anaphylactic reactions are rarely seen in relation to tuberculin testing. Such reactions have not been seen in the previous phase 1a and 1b trial with the rdESAT-6 skin test; however the necessary treatment for an anaphylactic reaction must be accessible.

A vasovagal reaction to the Mantoux injection can occur.

The rdESAT-6 + rCFP-10 skin test reagent must be injected intradermally into the superficial layer of the dermis by study staff with training and experience in the Mantoux testing technique.

9.1.11. Concomitant medication

Concomitant medication considered necessary for the volunteer during the course of the trial should be recorded in the concomitant medication pages of the case record form (CRF) of the volunteer.

Volunteers should omit to use the following medications or live vaccines during the trial as they are all known to act as immune modulators and are therefore likely to compromise the skin test results:

- any *live* vaccine
- immunoglobulin
- systemic corticosteroids
- methotrexate
- azathioprine
- cyclosporine
- blood products

However if such medications are taken, they must be recorded on the concomitant medication pages of the CRF.

Intake of the above listed medications would normally not compromise the safety of the trial volunteers and would therefore not necessarily lead to withdrawal from the trial. Decisions on withdrawal will be at the discretion of the investigator. See Section 8.4 for medications and live vaccines that are listed under exclusion criteria. Individuals who have taken these medications ≤ 3 months or have been vaccinated with a live vaccine 6 months PRIOR to entry in the trial should NOT proceed to Visit 2 - Inclusion Visit.

9.1.12. Investigational events

**Visit No.**	**1[2]**	**2**	**3**	**4**		**5**	**6/Final Visit[3]**	**Term Visit[4]**
**Group[1]**	**all**	**all**	**all**	**A/C**	**B/D**	**A/B/ C/D**	**all**	**all**
**Time of Visit** hours (h) / days (d)	**0 to -28d**	**Day 0[2]**	**T1 +72h**	**T1 +42d**	**T1 +84d**	**T2 +72h**	**T1/T2 +28d**	**Term Visit**
Verbal/written information to the volunteer	×							
Collect Signed Consent Form - allocate Subject No.	×							
Demography	×							
Medical history incl. interview on previous exposure to tuberculosis with recording of BCG and TST history	×							
Medical examination[5]	×						×[12]	×[12]
Blood sample (central lab.)[6]	×						×	×
Urine sample (central lab.)[7]	×						×	×
In-/exclusion criteria	×	×						
Inclusion		×						
Oral temperature[8]		×		×	×			
Pregnancy test	×	×		×	×			
First (T1) and second (T2) rdESAT-6 + rCFP-10 skin testing + observation 1 hour after injection[9]		**T1**		**T2**	**T2**			
Hand out diaries and a thermometer (only at visit 2)[10]		×		×	×			
Blood sample for QuantiFERON ® test[11]	×			×	×		×	×
Measurement of local reaction and digital photo of injection site			×			×	×[13]	×[13]
Adverse events		×	×	×	×	×	×	×
Concomitant medication		×	×	×	×	×	×	×
Trial completion							×	×

1) There are a total of 5 groups (A, B, C, D and E). In Groups A, B, C and D two injections (T1 and T2) are given. In Group E only one injection is given (T1)

2) The screening visit (Visit No. 1) takes place up to 28 days before the Inclusion visit (Visit No. 2)

3) For Groups A, B, C and D, Visit 6 is performed 28 days after T2. For Group E, Visit 6 is performed 28 days after T1

4) A termination visit should be performed for volunteers who drop out or are withdrawn from the trial

5) General medical examination

6) Blood samples for: Leukocytes (WBC) (- eosinophils, - basophils, - neutrophils, - lymphocytes, - monocytes), Platelets (Plts), Red blood cells (RBC), Haemoglobin (Hb), C-reactive protein (CRP), Glucose - random (GLU), Total bilirubin, Alkaline phosphatase (ALP), Aspartate transferase (AST), Alanine transferase (ALT), Albumin (Alb), Creatinine, Potassium (K^+), Sodium (Na^+) at Visits 1 and 6. HIV, HBV and HCV only at Visit 1)

7) Urine sample for: glucose and protein

8) Body temperature will be measured orally and must be below 38.0°C at Visit 2 and Visit 4

9) T1 = 1st rdESAT-6 + rCFP-10 test; T2 = 2nd rdESAT-6 + rCFP-10 test

10) Diary 1 is handed out on T1 day. Diary 2 is handed our on T2 day. The diaries are reviewed and collected at the following visits.

11) Blood sample for measuring *in-vitro* Interferon-γ response upon ESAT-6 stimulation with the QuantiFERON®-TB Gold In Tube test

12) Volunteers with a positive sensitisation reaction or a positive QuantiFERON test result will be asked to return for a final check 6 months after the last visit in the trial.

13) A digital image will only be taken if there are visible signs of a reaction to the rdESAT-6 + rCFP-10 tests

9.1.13. Allocation of volunteers to study groups

After the volunteer has been found eligible for the trial at the inclusion visit he/she is allocated to the first (not already occupied) study group with an acceptable time schedule (in the order A, B, C, D, E).

9.1.14. Assessment of safety

For safety reasons there will be at least 1 hour before the next volunteer is given an injection of the rdESAT-6 + rCFP-10 skin test reagent. All adverse events will be assessed and reported by the investigator, as specified in Section 11. A detailed description of any local or systemic adverse events will be made in the CRF.

In case of local adverse events, additional images, if applicable, will be made of both the RIGHT (All Groups) and the LEFT (Groups A, B, C and D) arm's injection sites at the time of occurrence. The course of any adverse events will be documented by sequential descriptions and, if applicable, by making digital images until the events have completely disappeared. Erythema ≥ 20 mm will be regarded as a local adverse reaction.

The volunteers will be given a diary after the first (All Groups) and the second (Groups A, B, C and D) rdESAT-6 + rCFP-10 test where all adverse events (local and systemic) should be recorded. The volunteers should record all adverse events (if any) and an oral temperature daily one week after the FIRST injection (All Groups) and two weeks after the SECOND injection (Groups A, B, C and D). At the following visits the recorded adverse events will be transferred by the investigator to the CRFs.

In a previous phase 1a clinical trial conducted at Leiden University Medical College, the Netherlands, the rdESAT-6 (only) skin test was investigated at 4 different dose levels (0.01 µg, 0.1 µg, 1 µg and 10 µg) in a group of healthy volunteers and later at 3 different dose levels (0.01 µg, 0.1 µg and 1 µg) in a group of previously treated TB-patients.

No toxicity signs were seen in the 4 groups of healthy volunteers. In previous TB patients minor local adverse reactions were seen in the groups of 0.01µg and 0.1µg rdESAT-6. Increasing the dose to 1µg responses were seen in most subjects and there was a higher frequency and intensity of local adverse reactions. There were also general complaints in this group e.g headache, however, with uncertain relationship to the trial product.

In the later phase 1b clinical trial conducted at Rigshospitalet, Copenhagen to assess the sensitisation risk of repeated injections of a fixed dose of 0.1 µg rdESAT-6 (only) at time intervals of 28, 56 and 112, it was documented that the repeated injections of the rdESAT-6 skin test could be given safely.

As previously mentioned Reece et al [33] recently suggested that CFP-10 may trigger "Tuberculin shock". He found that 5/10 guinea pigs died 6-36 hours after the skin testing with CFP-10, if the test was done 6 weeks after IV infection. No deaths were observed if the skin testing was done after 4 weeks or earlier. A more likely explanation is that the testing and the deaths were coincidental as the guinea pigs after 6 weeks of infection were at a late stage of disease. In a repeat study No. F1137 [40] done at SSI

with an infection period of 6 weeks before testing, 3/30 (10%) animals died or were about to die from tuberculosis even before skin testing. The same number of animals died after skin testing with rCFP-10 or PPD. It is likely that any immunological reagent at this time of disease will be able to induce a shock-like syndrome in the animals.

Non clinical toxicity studies performed in 2008 concluded that repeated subcutaneous injections of 10µg rdESAT-6 + rCFP-10 were safe in rats.

Based on the non clinical pharmacology and toxicity studies and two phase I clinical trials with rdESAT-6 (alone), it is expected that administration of the rdESAT-6 + rCFP-10 skin test by the Mantoux injection technique in doses between 0.01 µg and 1.0 µg only will expose the volunteers to MINIMAL RISKS, such as local reversible adverse reactions at the injection sites. The rdESAT-6 + rCFP-10 test is not likely to give rise to systemic adverse reactions.

9.1.15. Assessment of sensitisation

In the previous phase 1a and 1b clinical trials investigating the rdESAT-6 (alone) skin test reagent both redness and induration were observed as delayed type hypersensitivity reactions. The result of the classical tuberculin skin test is, however, by consensus expressed as millimetres induration only and induration (only) will therefore be regarded as the result to the new rdESAT-6 + rCFP-10 skin test reagent. Redness will in this present clinical trial be measured but will only be regarded as an exploratory value.

Delayed-type hypersensitivity (DTH) reactions will be measured independently by two experienced members of the study staff team and then compared.

In case of a discrepancy between the corresponding values of both study staff members, consensus will be sought by examining the reactions together. Only the consensus diameter will be recorded in the CRF.

On the day of the injections, the injection sites of the test substances will be marked with four dots on the direct proximal, distal arm with a black marker pen at a distance of at least 3 cm from the injection sites. This is to avoid later confusion about the precise location of the injection sites of the skin tests and to allow orientation on the digital images. The FIRST rdESAT-6 + rCFP-10 test (All Groups) will be placed on the RIGHT forearms of the volunteers and the SECOND rdESAT-6 + rCFP-10 test (Groups A, B, C and D) will be placed on the LEFT forearms of the volunteers.

Induration is measured by palpation of the injection sites from lateral to central on all sides. Because the study staff will make independent measurements of the diameters of induration, the borders will not be marked on the skin.

Redness is the visible red or pink discoloration of the skin around the injection sites. The border is assessed visually. Because the study staff will make independent measurements, the staff will not mark the borders on the skin.

Digital images of the injection site on the RIGHT forearm will be made for ALL volunteers at visit 3 and on the LEFT forearm at visit 5 in Groups A, B, C and D. A digital image will only be taken on the LEFT forearm at visit 6 if there are visible signs

of a local reaction to the rdESAT-6 + rCFP-10 test. A digital image of the RIGHT injection site at the last visit for Group E will only be taken if there are visible signs of a local reaction.

Next to the arm, a label will be placed indicating the subject number, visit number and, finally, if it is the FIRST or SECOND injection with the rdESAT-6 + rCFP-10 test. A ruler with millimetre indications will be placed at least 3 cm from the injection site.

Injection of the rdESAT-6 + rCFP-10 skin test reagent is intended to identify latent tuberculosis infection by the formation of a local erythema and induration at the injection site 48 h - 96 h after the injection (positive reaction). No reaction is expected to occur in uninfected persons after a single injection. In the present study a skin reaction after the first injection will be regarded as a sign of latent infection or an adverse reaction. We will investigate if a positive skin reaction occurs after a second injection of rdESAT-6 + rCFP-10 given to healthy individuals at an interval of 6 or 12 weeks. In case such a response appears it may be regarded as a sensitisation reaction induced by the preceding injection. Such a false positive skin reaction appears similar to a positive skin reaction in a TB infected person. To the individual the response does not represent a safety issue, but it is an undesired property by the reagent.

The sensitisation risk of rdESAT-6 + rCFP-10 will be evaluated by assessing the diameter of induration of the injection site 72 hours after the injection of the SECOND rdESAT-6 + rCFP-10 dose (i.e reading in millimetres). An induration of ≥ 6mm will be regarded as a possible sensitisation reaction.

In addition, the sensitisation risk will be evaluated by comparing and assessing the *in vitro* interferon -γ response from blood samples taken at the screening visit, prior to administration of the second rdESAT-6 + rCFP-10 dose and 28 days after the second rdESAT-6 + rCFP-10 dose using the QuantiFERON®-TB Gold In Tube test. An IFN-γ response of ≥ 0.35 IU/mL will be regarded as a possible sensitisation reaction.

Volunteers with a positive sensitisation reaction or a positive QuantiFERON test result will be asked to return for a final check 6 months after the last visit in the trial to rule out a tuberculosis infection.

9.1.16. Collection and handling of blood samples

Venous blood samples will be obtained by the standard techniques at Rigshospitalet using the Vacutainer® system. All local guidelines and safety measures of Rigshospitalet with regard to blood samples will be followed. Tubes will be labelled with Rigshospitalet's 'identification stickers' with a unique number and a unique bar code identifying the volunteer from whom the sample was taken and the date the sample was taken.

Standard laboratory forms will be used and print-outs from the laboratory will be signed and initialized by investigator. In case of out of range values assessed by the investigator as not clinically significant, the investigator will write NS (or a similar abbreviation) at the individual value and date and sign the value (with initials).

At each blood drawing 11 mL blood will be drawn for the safety tests, 3 mL blood for the QuantiFERON test and 7 mL blood (only at screening) for the HIV, HBV and HCV tests.

Blood samples to the Department of Clinical Chemistry will be transported according to standard procedures at Rigshospitalet. Blood samples for the QuantiFERON tests will be transported to the Mycobacteria Laboratory at Statens Serum Institut by taxi.

9.1.17. Laboratory assays

The routine laboratory assays will all be performed at the Department of Clinical Biochemistry at Rigshospitalet according to standard procedures.

The QuantiFERON®-TB Gold In Tube test will be performed at the Department of Mycobacteria at Statens Serum Institut according to standard procedures.

Out of range blood test results for the routine chemistry and haematology laboratory assays will be assessed by the investigator. If the investigator assesses that the values are NOT clinically significant the volunteer is eligible for inclusion.

9.1.18. Time schedule

The documents to the EC and The Danish Medical Agency (Lægemiddelstyrelsen) are planned to be submitted in September 2008.

The first patient's first visit (FPFV) is planned to be in November 2008.

The last patient's last visit (LPLV) is planned to be in April 2009.

10. Ethical Aspects

Tuberculosis continues to be a major cause of morbidity and mortality throughout the World as being one of the most important fatal infections of human beings, with 9 million new cases every year and an estimated 2 million deaths in 2004. [1].

To control the disease fast and accurate diagnosis is very important. The new diagnostic tool to be investigated in this phase 1 clinical trial is a traditional tuberculin test skin test but with two new antigens, rdESAT-6 and rCFP-10, as the active ingredients.

Participation in this clinical trial is voluntary, and the volunteer may choose to leave the trial at any time without any specific reason.

Before entering the trial, the volunteer will be informed (verbally and in writing) that there is a risk of unexpected adverse reactions, and that this risk has to be taken into account when considering participation. Detailed information about all trial procedures and the inconveniences these might pose on the volunteer will be explained as well. The volunteer will be informed that he or she is welcome to bring a third party to receive the information. A brochure from the Central Ethical Committee ("Før du beslutter dig" – see appendix 8) giving general information on the participation in clinical trials will be given to the volunteers before participation.

In 2005/2006 the rdESAT-6 (only) skin test synthesised in *Lactococcus lactis,* was administered to humans for the first time in a phase 1a clinical trial conducted at Leiden University Medical College, the Netherlands. The aim of this first phase 1 trial was to investigate the safety and the diagnostic potential of the rdESAT-6 skin test. The rdESAT-6 skin test was investigated at 4 different dose levels (0.01 µg, 0.1 µg, 1 µg and 10 µg) in a group of healthy volunteers and later at 3 different dose levels (0.01 µg, 0.1 µg and 1 µg) in a group of previously treated TB-patients.

No toxicity signs were seen in the 4 groups of healthy volunteers. In previous TB patients minor local adverse reactions were seen in the groups of 0.01 µg and 0.1 µg rdESAT-6. In the group of 1µg responses were seen in most subjects and there was a higher frequency and intensity of the local adverse reactions. There were general complaints in this group of e.g headache, however, with uncertain relationship to the trial product.

In the succeeding phase 1b clinical trial (TESAT-02) investigating the sensitisation risk of repeated administration of the rdESAT-6 (only) skin test reagent; it was documented that the repeated injections of the rdESAT-6 skin test could be given safely.

The trial participants in this current phase 1 trial will all be healthy volunteers ≥ 18 years and ≤ 55 years of age.

Individuals with ongoing conditions or medical histories which might make them more vulnerable to the procedures undertaken in this clinical trial or which might interfere with the performance of the skin test are excluded from participation. These are as follows:

- Haemophilia or other coagulation disorder (due to the risk from repeated blood drawings)
- Has been in treatment with a product which is likely to modify the immune response within 3 months prior to the day of inclusion
- Has been vaccinated with a live vaccine within 6 months prior to the day of inclusion
- Has a known congenital or acquired immune defiency
- Has a disease affecting the lymphoid organs (e.g Hodgkin's disease, lymphoma, leukaemia, sarcoidosis)
- Has a viral or bacterial infection that might affect the cell mediated immune response at inclusion

Non clinical toxicity studies performed in 2008 concluded that repeated subcutaneous injections of 10µg rdESAT-6 +rCFP-10 were safe in rats.

Based on the non clinical pharmacology and toxicity studies of the rdESAT-6 + rCFP-10 and two phase I clinical trials with rdESAT-6 (alone), it is expected that the administration of the rdESAT-6 + rCFP-10 skin test by the Mantoux injection technique in doses between 0.01 and 1.0 µg only will expose the volunteers to MINIMAL RISKS, such as local reversible adverse reactions at the injection sites. The rdESAT-6 + rCFP-10 test is not likely to give rise to systemic adverse reactions.

Detailed information on the planned number and amount of blood samples will be part of the verbal and written information.

In conclusion, it is anticipated that there is a reasonable balance between the risks and the inconveniences to which the volunteers will be exposed and the benefits they might obtain as a result of the increased medical attention which is part of the clinical trial, i.e. medical examinations and laboratory investigations.

10.1. Payment of Volunteers

Trial volunteers assigned to groups A-D are paid 1800 DKK (liable to pay taxes) at the end of the trial (i.e. on completion of Visit 6) as compensation for the number of blood samples they have given and for inconveniences caused by the intradermal administration of the skin tests. In addition the volunteer will be paid 200 DKK (liable to pay taxes) as compensation for transport expenses which will be paid (in cash) after the completion of each visit. The volunteers sign for the receipt of all payments.

A healthy volunteer assigned to groups A-D and who completes the entire trial will be paid a total of:
1800 DKK + (6 × 200 DKK) = **3000 DKK**

Trial volunteers assigned to group E are paid 1200 DKK (liable to pay taxes) at the end of the trial (i.e. on completion of Visit 6) as compensation for the number of blood samples they have given and for inconveniences caused by the intradermal administration of the skin tests. In addition the volunteer will be paid 200 DKK (liable to pay taxes) as compensation for transport expenses which will be paid (in cash) after the completion of each visit. The volunteers sign for the receipt of all payments.

A healthy volunteer assigned to group E and who completes the entire trial will be paid a total of:
1200 DKK + (4 × 200 DKK) = **2000 DKK**

A budget is part of the agreement signed by the Department of Infectious Diseases at Rigshospitalet and SSI.

10.2. Data Safety Monitoring Plan

The present clinical trial is expected to expose the trial volunteers to minimal risks only. The principal investigator takes the responsibility for the safety of the volunteers in the daily clinical practice. However, the principal investigator might need advice in certain critical clinical situations and, therefore, a Data Safety Monitoring Board has been established.

The Trial Safety Board consists of:

Dr. Sandra M. Arend (head of the Data Safety Monitoring Board, independent),
Dr. Thomas Benfield (member of the Data Safety Monitoring Board, independent),
Dr. Pernille Ravn (member of the Data Safety Monitoring Board, independent),
Principal investigator Åse Bengård Andersen (Rigshospitalet),
Responsible Medical Advisor Trine R. Nielsen (SSI)
Study director Pernille Nyholm Tingskov (SSI).

The Data Safety Monitoring Board will, when needed, as judged by the principal investigator, evaluate critical events that occur during the trial. The independent Data Safety Monitoring Board members are all medical experts in the Netherlands and in Denmark.

When the last volunteer has completed **Visit 3** in the respective groups A to D the principal investigator will go through all relevant safety information and arrive at a recommendation as to whether or not to proceed with the SECOND injections in the respective groups. Furthermore, safety information available at **Visit 3** in group A (0.01 µg/0.1 mL) will be evaluated before group C (0.1 µg/mL) volunteers can be given their first injection, as well as safety information available at **Visit 3** in group C (0.1 µg/mL) will be evaluated before any injection is given to group E (1.0 µg/mL) volunteers.

In a brief written statement the principal investigator will give a review of reported adverse events and she will give her recommendations in respect to giving the SECOND injections and in respect to initiating the FIRST injections at the subsequent dose level. The principal investigator will also decide if a DSMB meeting is needed.

This brief written statement will be provided by e-mail to the study director, to all investigators in the trial, and to the Data Safety Monitoring Board members by e-mail as soon as possible and no later than 7 days after the last patient's Visit 3 in groups A, B, C and D. The study director will make sure that Trine R. Nielsen (medically responsible at SSI) and Anders Mørup Jensen (statistically responsible at SSI) are kept informed at all times.

At most occasions e-mail correspondence is expected to be sufficient for discussions and for reaching conclusions. If discussions take place via e-mail, the study director, all investigators in the trial and all Data Safety Monitoring Board members should always receive copies, so that these persons are well informed at any time.

11. Adverse Events

This section reviews the procedures for recording and reporting of adverse events in the trial. Relevant definitions and terms are listed. Furthermore, the procedures for immediate reporting of serious adverse events to SSI and for expedited reporting to the competent authorities and to the local ethics committee are described.

11.1. Definitions and terms

All definitions in the following are in accordance with the ICH E2A guideline [21].

Adverse Event (AE)
Any untoward medical occurrence in a patient or a volunteer participating in a clinical investigation and receiving a pharmaceutical product, which does not necessarily have a causal relationship with this product.

Adverse Reaction/Adverse Drug Reaction/Suspected Adverse Reaction (AR/ADR/SAR)
Any untoward and unintended response to an investigational product related to any dose administered. The terms 'Adverse Reaction', 'Adverse Drug Reaction and 'Suspected Adverse Reaction' are the same thing (in practice), and imply that there is a

suspected relationship between the event and the trial product. In practice this means that there is evidence or arguments that suggest a causal relationship, i.e. a relationship cannot be ruled out.

Seriousness criteria
A serious adverse event or reaction is any untoward medical occurrence that at any dose:

– results in death
– is life-threatening
– requires in-patient hospitalisation or prolongation of hospitalisation
– results in persistent or significant disability/incapacity
– is an important medical condition

NOTE: The term 'life-threatening' in the above definition refers to an event during which the patient was at risk of death at the time of the event, it does not refer to an event which hypothetically could have caused death, had it been more severe.
If a SAR (suspected adverse reaction) is serious it is a 'serious suspected adverse reaction' (SSAR).

Unexpected adverse reaction
An adverse drug reaction, the nature or severity of which is not consistent with the applicable product information (e.g., the Investigator's Brochure).

If a SSAR (serious suspected adverse reaction) is unexpected it is a 'Suspected Unexpected Serious Adverse Reaction' (SUSAR).

Immediate reporting
The investigator's rapid reporting of a serious *AE/AR/ADR/SAR* to SSI (reporting within 72 hours of the investigator's first knowledge of the *AE/AR/ADR/SAR)*.

Expedited reporting
SSI's rapid reporting of a suspected unexpected serious adverse reaction (SUSAR) to the concerned competent authorities and the ethics committees. Only, SUSARs must be reported not SSARs. In Demark, the ethics committees only want annual update reports (i.e. no expedited reporting to the ethics committees is required in this trial).

Fatal or life-threatening, SUSARs occurring in the trial qualify for very rapid reporting, and the Danish Medicine Agency (Lægemiddelstyrelsen) should be notified (e.g., by telephone, facsimile transmission, or in writing) as soon as possible but no later than 7 calendar days after the sponsor's first knowledge of the event, followed by a complete a report within 8 additional calendar days.

SUSARs, which are not fatal or life-threatening, must be submitted as soon as possible but no later than 15 calendar days after the sponsor's first knowledge of the case meeting the criteria for expedited reporting.

11.2. Standard reporting of adverse events

The investigator is responsible for the recording of all reported adverse events in the Adverse Event Forms of the CRFs during the visits. The investigator must use the following terms:

The **causal relationship** between an adverse event and the trial tests is assessed using the following terms:

- Not related
- Possible
- Probable
- Certain

The **intensity** of an adverse event is assessed using the following terms:

- Mild (i.e. easily tolerated)
- Moderate (i.e. sufficient to interfere with daily activities)
- Severe (i.e. sufficient to prevent normal activity)

The **outcome** of an adverse event is assessed using the following terms:

- Fatal
- Not yet recovered
- Recovered with sequelae
- Recovered without sequelae
- Unknown

NOTE: If an adverse event is still ongoing at the last visit, it must be followed by the investigator until it has resolved or stabilised.

The **seriousness** of an adverse event is assessed by answering the following questions:

- Did the event result in death?
- Has the event been or is the event life-threatening?
- Has the event required inpatient hospitalisation or prolonging of hospitalisation?
- Has the event resulted in significant or persistent disability or incapacity?
- Is the event medically important

To ensure no confusion or misunderstanding of the difference between the terms "serious" and "severe," which are not synonymous, the following note of clarification is provided:

The term "severe" is often used to describe the intensity (severity) of a specific event (as in mild, moderate, or severe myocardial infarction); the event itself, however, may be of relatively minor medical significance (such as severe headache). This is not the same as "serious," which is based on patient/event outcome or action criteria usually associated with events that pose a threat to a patient's life or functioning. Seriousness (not severity) serves as a guide for defining regulatory reporting obligations.

11.3. Expedited reporting of adverse events

The investigator is responsible for the immediate reporting (within 72 hours) to SSI of all serious adverse events (SSARs and SUSARs).

The investigator should always exercise medical and scientific judgement in deciding whether immediate reporting is appropriate.

The investigator should fill in the CIOMS form (see Appendix 1) and fax it to the Department of Regulatory & Medical Affairs at SSI within 72 hours of his/her first knowledge of a serious adverse reaction/event.

The initial report should be followed by follow-up reports (using the same form) if additional important information becomes available.

SSI will ensure that expedited reporting to the competent authorities within the required time lines.

Furthermore, SSI is responsible for submitting annual safety update reports of serious adverse events (SUSARs and SSARs) to the competent authorities and to the ethics committees (or as required by the competent authorities or ethics committees).

Post study events:
Although such information is not routinely sought or collected by SSI, serious adverse events that occurred after the patient has completed the trial will possibly be reported by the investigator to the SSI. Such cases will be reported expedited to the competent authorities if they meet the criteria for expedited reporting specified above.

12. Data Management and Statistical Analysis

12.1. General considerations

The Biostatistics Unit at SSI will be responsible for data management and statistical analysis of the trial data.

12.2. Data management

Clinical data is collected in the CRFs. The CRFs are in English. The volunteers' diaries are in Danish and will be given to the volunteers on the days of skin testing. Information on adverse events noted in the diaries are transferred to the CRF by the investigator. The diaries are considered supportive for the investigators when filling in the CRFs, i.e. only relevant information from the diaries will be translated and transferred to the CRFs by the investigator. The diaries will not be sent to the sponsor's site but will remain filed at the investigator's site and filed with the investigator's copy of the CRFs.

The haematology and urinalysis data from the central lab. at Rigshospitalet will be submitted to the sponsor as paper print-outs.

The data from the QuantiFERON TB Gold in Tube test, analysed at SSI, will be submitted to the sponsor as paper print-outs.

Data from the CRFs, the central lab. at Rigshospitalet, and the QuantiFERRON results will be entered into SAS data sets and checked for consistency and plausibility by custom-made SAS programs. All ambiguous or implausible data items will be resolved by data queries to the investigators.

12.3. Clean file procedures

At the end of the trial, when the data entry from the CRFs and the laboratory measurements have been validated, the SAS data sets reach clean file status. Identical copies on CDs of the SAS data sets will then be made. One CD will be archived in the trial master file. The other CD will be archived at the Biostatistics unit.

The statistical analysis reported in the integrated clinical trial report will be based on the clean file data.

12.4. Analysis populations

All volunteers who have received at least one injection with the test substance will be included in the analysis.

12.5. Statistical methods

Because of the limited number of subjects included in the trial the statistical methods will mostly be descriptive.

All reported AEs, IFN-γ results, induration measurements and standard (clinical/chemical) laboratory measurements will be listed.

IFN-γ results, induration measurements and the standard laboratory measurements will be presented as plots.

12.6. Sample size determinations

The sample size is primarily based on practical issues and not statistical considerations.

Assume that the risk of sensitisation (defined as induration $\geq$ 6 mm **or** IFN-$\gamma \geq$ 0.35 IU/mL as measured by QuantiFERON® TB Gold after the second injection) increases with dose and for a fixed dose of rdESAT-6 + rCFP-10 is higher if the two injections are given with a 6 week interval than with a 12 week interval.

If none of the 40 subjects in Groups A, B, C and D show evidence of sensitisation, not only the 10 subjects in that particular combination of dose and injection interval, but also those with a higher dose and those with a shorter injection interval can be taken into consideration in a conservative calculation of a one-sided upper 95% CI for the risk of sensitisation.

With an effective sample size n (as defined above) the conservative one-sided upper 95% CI is calculated as [0 , $1-(1-0.95)^{(1/n)}$].

Conservative 95% one-sided CI for risk of sensitisation

	Time between injections			
	6 weeks		12 weeks	
Dose	Effective sample size	95% CI	Effective sample size	95% CI
0.01 µg	20	(0.00, 0.14)	40	(0.00, 0.07)
0.1 µg	10	(0.00, 0.26)	20	(0.00, 0.14)

13. Good clinical practice considerations

The present phase I clinical trial is performed with the *in-vivo* diagnostic skin test, rdESAT-6 + rCFP-10 in humans. rdESAT-6 and rCFP-10 are both fermented in recombinant strains of *Lactococcus lactis* and purified separately and are, therefore, regarded as a biotechnologically-derived pharmaceutical.

The internationally recognised guidelines ICH S6 [16], ICH M3 [17] and ICH S7A [22] were used for the design of the pre-clinical study programme of rdESAT-6 + rCFP-10. However, as these guidelines are aimed at traditional pharmaceutical products, some adjustments were needed due to the special nature of rdESAT-6 + rCFP-10.

The ICH guideline, ICH Topic E 6 [18] and ICH Topic E 2A [21] were followed when designing this clinical trial. Furthermore the following documents were consulted for advice:

Directive 2001/20/EC and 2005/28/EC of the European Parliament (including underlying guidances) [19, 20]

Guideline on strategies to identify and mitigate risks for first-in-human clinical trials with investigational medicinal products. EMEA/CHMP/SWP/28367/07; London 19 July 2007 [35]

"Bekendtgørelse om god klinisk praksis I forbindelse med kliniske forsøg med lægemidler på mennesker , Nr 744 af 29. juni 2006" [23]

"Bekendtgørelse om kliniske forsøg med lægemidler på mennesker. Nr 295 af 26. april 2004 " [24]

"Bekendtgørelse om ændring af bekendtgørelse om kliniske forsøg med lægemidler på mennesker. Nr 903 af 18. august 2006" [25]

"Vejledning til ansøgning om tilladelse til kliniske forsøg med lægemidler på mennesker, Lægemiddelstyrelsen oktober 2006" [26]

"Vejledning om anmeldelse m.v. af et biomedicinsk forskningsprojekt til det videnskabsetiske komitésystem, 1. september 2007" [27]

13.1. Declaration of Helsinki

This clinical trial will be conducted in accordance with the principles of the Declaration of Helsinki (see Appendix 2).

13.2. Volunteer information and informed consent

Healthy volunteers will mainly be recruited from the University of Copenhagen. Advertisements/posters will be objective and balanced and will be approved by the Ethics Committees, Region of Copenhagen ("De videnskabsetiske komitéer for Region Hovedstaden").

Potential volunteers are informed about the trial both verbally and in writing. The information is provided by trained staff in an objective way, describing both advantages and disadvantages of participation in the trial. The volunteer will be informed that he or she is welcome to bring a third party to receive the information.

After the verbal information, the 'Paticipant Information Sheet' (see Appendix 3) and a brochure from the Central Ethical Committee ("Før du beslutter dig") giving general information on the participation in clinical trials, will be given to the volunteers.

The informed volunteer is given reasonable time to consider whether he/she wishes to participate.

13.3. Independant Ethics Committee submission and approval

An application file will be submitted for approval to the Ethics Committee: ("De videnskabsetiske komiteer for Region Hovedstaden", Regionsgården, Kongens Vænge 2, 3400 Hillerød, Tlf. 4820 5585).

The inclusion of volunteers in the clinical trial will start AFTER the trial has been approved by the ethics committee.

13.4. Regulatory health authority submission and approval

An application file will be submitted for approval to the regulatory authorities in Denmark: the Danish Medicine Agency (Lægemiddelstyrelsen, Axel Heides Gade 1, 2300 København S). Only after approval by **both** the Ethics Committee ("De videnskabsetiske komiteer for Region Hovedstaden") and the Danish Medicine Agency the clinical trial will be initiated.

13.5. Volunteer data protection

The investigators are responsible for keeping a list (screening log) of all volunteers in the trial, including the subject numbers, full names and last addresses known, etc.

SSI will receive information on subject numbers, sex and date of birth only. This information will be traceable to the screening log.

Furthermore, the volunteers are informed in writing that the results will be stored and analysed by computer and that confidentiality will be maintained. Relevant Danish laws will be followed in maintaining confidentiality [38, 39].

The volunteers are also informed (in writing) that authorised persons from the investigator's site, from the sponsor's site, or from the regulatory authorities may need to review his/her hospital records in relation to the clinical trial. By signing the informed consent form (see Appendix 4) the volunteer accepts these conditions.

Statens Serum Institut will according to local regulations inform "Datatilsynet" about the clinical trial.

13.6. Investigator's responsibilities

The Investigator is responsible for the conduct of the clinical trial in accordance with the protocol, Good Clinical Practice (GCP) [18, 19, 20] and relevant Danish regulations [23, 24, 25]. The Investigator is, moreover, responsible for the proper reporting of all adverse events according to the procedures described in this protocol in Section 11.

If the principal investigator delegates her responsibilities to another member of the staff this must be approved by SSI and confirmed in writing.

13.7. Curricula vitae and log(s) of staff

Before initiation of the trial, current signed and dated curricula vitae in English for the principal investigator, other investigators, other significant staff at the trial site and staff at the sponsor's site (SSI) must be collected. Staff participating in the clinical trial at the trial site and at the sponsor's site will, furthermore, be listed in log(s) of staff. For trial monitors, data managers, and other persons who are allowed to make entries in the CRFs or in other trial related documents, signatures and initials must also be listed in the log(s) of staff. The log of staff will be filed in the Investigator's file at the clinical trial site and in the Trial Master File at SSI.

13.8. Indemnity statement

Prior to the inclusion of the first volunteer in the clinical trial, SSI will present a signed Indemnity Statement to all investigators participating in the clinical trial (see Appendix 5).

13.9. Training

Before the inclusion of the first volunteer in the trial, it will be ensured that all study staff is adequately qualified. The study director will if necessary perform training sessions covering general GCP issues, for example: safety procedures, reporting of Suspected Unexpected Serious Adverse Reactions (SUSARs), information of volunteers, handling of test products etc. If training is needed in relation to the Mantoux testing technique, tuberculosis, etc., the principal investigator will perform these sessions. All performed sessions will be documented in the CVs of the investigators and the staff members performing the Mantoux testing etc.

13.10. Monitoring

The clinical trial monitor from SSI will make regular visits to the investigational site at Rigshospitalet. Together with the study staff at the site, the monitor will, for example, check the following:

- That the protocol is being followed
- That facilities and staffing remain acceptable
- That the CRFs are being correctly filled in
- That the CRFs are in accordance with source data
- That the clinical supplies (especially the test products) can be accounted for
- That the test products are stored properly
- That the Investigator's File is being kept in proper order

All visits to the investigational site are documented. Any query is discussed and resolved with the investigator or relevant study staff.

13.11. Audit and inspection

The investigator must give access to personnel from SSI for the conduct of audits. Regulatory authorities must at all times be allowed access to conduct inspections as well. Auditors and inspectors must have access to all trial related documents, including the Investigator's File and the volunteers' personal medical records.

13.12. Definition of source data

Source data is defined as all information in original records and certified copies of original records necessary for the reconstruction and evaluation of the clinical trial. A document, which identifies the sources of data will be prepared before initiation of the trial. In the following examples of source data are listed:

- EC approval documents
- Approval from the Danish Medicine Agency (Lægemiddelstyrelsen)
- Signed informed consent forms

- Nurse notes (i.e. for data either not recorded in the CRF or for data not recorded directly in the CRF)
- The CRF for data recorded directly in the CRF
- Dispensing of test product and accountability information in logs
- The screening log for e.g. subject number, full name and address

13.13. Archiving of essential documents

It is the responsibility of the principal investigator and SSI to maintain the essential documents as described in the ICH guidelines for at least 15 years after the termination of the trial (i.e. after the last patient's last visit).

The principal investigator is responsible for archiving (at least) of the following documents:

- Signed Consent Forms of all volunteers
- Screening log
- Investigator's copy of the CRFs
- Diaries
- Completed CIOMS forms of Suspected Serious Adverse Events
- All source documents or certified copies of source documents

The completeness of the Investigator's File should be documented by SSI as part of the termination monitoring visit.

14. Agreement and financial settlement

The agreement between the Investigational institution (Rigshospitalet) and SSI must be signed prior to inclusion of the first volunteer in the clinical trial. The agreement must clearly state the rights and obligations of the parties concerned and include a detailed financial settlement.

Furthermore, a written agreement will be made between the principal investigator and the laboratory at Rigshospitalet, regarding the safety laboratory analysis.

15. Insurance

SSI is the sponsor and manufacturer of the test products to be administered in this clinical trial. SSI carries a product liability insurance under a worldwide liability programme written by the New Hampshire Insurance Company, through insurance brokers Marsh A/S, Teknikerbyen 25, DK-2830 Virum, Denmark, as part of the worldwide Marsh insurance broker group. The policy covers claims arising from injury/injuries caused by trial medication used in clinical trials sponsored by the

company, if the trial medication has been used in accordance with the instructions given in the protocol. The insurance certificate is enclosed in Appendix 6. The insurance conditions will be explained in writing to the volunteers (see Appendix 3).

16. Confidentiality and disclosure

All CRFs, information and results generated by SSI, as well as information on product development, patented or not, including patent applications and manufacturing processes not previously published, are considered confidential and shall remain the sole property of SSI.

No data from the clinical trial may be published, presented or communicated, except to regulatory authorities or ethics committees, prior to the release of the internal clinical trial report, unless approved by SSI in writing. All investigators agree not to discuss externally or publish any result from the trial without the possibility of SSI to give comments for up to 90 days after receipt of the manuscript.

In the event of a publication the names of the authors and their order of appearance will be as specified in the agreement between the Investigational institution (Rigshospitalet) and SSI.

17. Changes to the protocol

The clinical trial procedures may be changed if the principal investigator and the study director agree to the changes. If the changes are substantial, both the Ethics Committee and Competent Authorities must approve the changes before they can be implemented. All substantial changes must be documented by protocol amendments and rewritten full protocols, if applicable.

18. References

[1] World Health Organisation. Global Tuberculosis Control: Surveillance, Planning, Financing. WHO Report 2006.Geneva Switzerland, (WHO/HTM/TB/2006.362) ISBN 92 4 156314 1

[2] Snider DE. Tuberculin test. Am Rev Respir Dis 1982; 125: 108-18

[3] Huebner RE, Schein MF, Bass JB. The tuberculin skin test.
Clin Infect Dis 1993; 17: 968-75

[4] Andersen P, Munck ME, Pollock JM, Doherty TM. Specific immune-based diagnosis of tuberculosis. Lancet 2000; 356: 1099-104

[5] Andersen P, Andersen AB, Sorensen AL, Nagai S. Recall of long-lived immunity to *Mycobacterium tuberculosis* infection in mice.
J Immunol 1995; 154: 3359-72

[6] Ulrichs T, Munk ME, Mollenkopf H, et al. Differential T-cell responses to *Mycobacterium tuberculosis* ESAT-6 in tuberculosis patients and healthy donors. Eur J Immunol 1998; 28: 3949-58

[7] Ravn P, Demissie A, Eguale T, et al. Human T-cell responses to the ESAT-6 antigen from *Mycobacterium tuberculosis.*
J Infect Dis 1999; 179: 637-45

[8} Johnson PD, Stuart RL, Grayson ML, Olden D, Clancy A, Ravn P, Andersen P, Britton WJ, Rothel JS. Tuberculin-purified protein derivative-,
MPT-64-, and ESAT-6-stimulated gamma interferon responses in medical students before and after M. *bovis* BCG vaccination and in patients with tuberculosis. Clin Diagn Lab Immunol 1999; 6: 934-37

[9] Lein AD, von Reyn CF, Ravn P, Horsburgh CR et al.Cellular immune responses to ESAT-6 discriminate between patients with pulmonary disease due to *M.avium* complex and those with pulmonary disease due to *M. tuberculosis.*Clin Diagn Lab Immunol 1999; 6: 606-609

[10] Doherty TM, Demissie A, Joseph O, Wolday D, Britton S, Eguale T, Ravn P, Andersen. J Clin Microbiol. 2002. 40 (2): 704-06

[11] Elhay MJ, Oettinger T, Andersen P. Delayed type hypersensitivity responses to ESAT-6 and MPT-64 from *Mycobacterium tuberculosis* in the guinea pig. Infect Immune 1998; 66: 3454-56

[12] Aggerbeck H, Madsen S.M. Safety of ESAT-6. Elsevier, Tuberculosis (2006) 86; 363-373

[13] Pai Madhukar, Kalantri Shriprakash, Dheda Keertan. New tools and emerging technologies for the diagnosis of tuberculosis: Part 1. Latent tuberculosis. Expert Rev. Mol. Diagn. 6(3), (2006). 2006 Future Drugs Ltd.

[14] Pai Madhukar, Riley Lee W, Colford John M Jr. Interferon-γ assays in the immunodiagnosis of tuberculosis: a systemic review. The Lancet, Infectious Diseases Vol 4 December 2004.

[15] Pai Madhukar, Gokhale Kaustubh, Joshi Rajnish, Dogra Sandeep, Kalantri Shriprakash, Mendiratta Deepak K., Narang Pratibha, Daley Charles L., Granich Reuben M., Mazurek Gerald H., Reingold Arthur L., Riley Lee W., Colford John M. Jr.. *Mycobacterium tuberculosis* Infection in Health Care Workers in Rural India. JAMA, June 8, 2005 – Vol 293, No. 22

[16] Note for guidance on pre-clinical safety evaluation of biotechnology derived pharmaceuticals. ICH Topic S 6. (CPMP/ICH/302/95)

[17] Note for guidance on non-clinical safety studies for the conduct of human clinical trials for pharmaceuticals. ICH Topic M 3 (M).CPMP/ICH/286/95, modification)

[18] Note for guidance on good clinical practice. ICH Topic E 6. (CPMP/135/95)

[19] Directive 2001/20/EC of the European Parliament and the council of 4 April 2001 on the approximation of the laws, regulations and administrative provision of the Member States relating to the implementation of good clinical practice in the conduct of clinical trials on medicinal products for human use. Official Journal L 121, 01/05/2001 P.0034 – 0044.

[20] Directive 2005/28/EC of the European Parliament of 8 April 2005 laying down principles and detailed guidelines for good clinical practice as regards investigational medicinal products for human use, as well as the requirements for authorisation of the manufacturing or importation of such products.

[21] Note for guidance on clinical safety data management: definitions and standards for expedited reporting ICH Topic E 2 A (CPMP/ICH/377/95)

[22] Topic S 7A. Safety pharmacology studies for human pharmaceuticals (CPMP/ICH/539/00) March 2000

[23] Bekendtgørelse om god klinisk praksis I forbindelse med kliniske forsøg med lægemidler på mennesker , Nr 744 af 29. juni 2006

[24] Bekendtgørelse om kliniske forsøg med lægemidler på mennesker. Nr 295 af 26. april 2004

[25] Bekendtgørelse om ændring af bekendtgørelse om kliniske forsøg med lægemidler på mennesker. Nr 903 af 18. august 2006

[26]Vejledning til ansøgning om tilladelse til kliniske forsøg med lægemidler på mennesker, Lægemiddelstyrelsen oktober 2006

[27] Vejledning om anmeldelse m.v. af et biomedicinsk forskningsprojekt til det videnskabsetiske komitésystem, 1. september 2007

[28] A. H van Pinxteren, Laurens et al. Diagnosis of Tuberculosis Based on Two Specific Antigens ESAT-6 and CFP10.Clinical and Diagnostic Laboratory Immunology, Mar 2000, p. 155-160

[29] Arend, Sandra M et al. Double blind randomized Phase 1 study comparing rdESAT-6 to tuberculin as skin test reagent in the diagnosis of tuberculosis infection. Tuberculosis (2007), doi: 10.1016/j.tube.2007.11.004.

[30] Points to Consider on the Evaluation of Diagnostic Agents. Committee for Proprietary Medicinal Products (CPMP/EWP/1119/98)

[31] Lillebæk T et al. Risk of sensitisation in healthy adults following repeated administration of rdESAT-6 skin test reagent by the Mantoux injection technique. (Submitted to Tuberculosis)

[32] Thierry-Carstensen B, Aggerbeck H and Jensen A.M. A phase I (first in man), souble blind, “within subject” randomized and controlled, dose-escalating clinical trial on the safety and the diagnostic potential of the rdESAT-6 skin test in the diagnosis of tuberculosis. Internal clinical study report date of issue 04-01-2008.

[33] Reece S.T., Stride N., Ovendale P., Reed S.G. et al. Skin test performed with highly purified Mycobacterium tuberculosis recombinant protein triggers tuberculin schock in infected guinea pigs. Infection and Immunity, June 2005, p. 3301-3306.

[34] Renshaw P. Lightbody K.L, Veverka V et al. Structure and function of the complex formed by the tuberculosis virulence factors CFP-10 and ESAT-6. The EMBO Journal, 2005, 24, 2491-2498

[35] Guideline on strategies to identify and mitigate tasks for first-in-human clinical trials with investigational medicinal products. Doc. Ref. EMEA/CHMP/SWP/28367/07; London 19 July 2007

[36] Skjøt, R.L.V., T. Oettinger, I Rosenkrands, P. Ravn, I. Brock, S. Jacobsen, P. Andersen. Comparative evaluation of low-molecular-mass T-cell antigens from *Mycobacterium tuberculosis* identifies members of the ESAT-6 family as immunodominant. Infect. Immun. 2000, 68: 214-220

[37] Elhay MJ, Oettinger T, Andersen P. Delayed type hypersensitivity responses to ESAT-6 and MPT-64 from *Mycobacterium tuberculosis* in the guinea pig. Infect Immune 1998; 66: 3454-6.

{38] Lovbekendtgørelse nr 95 af 7. februar 2008 – Sundhedsloven

[39] Lov nr 429 af 31. maj 2000 om behandling af personoplysninger

[40] Internal SSI Report F1137

SUSPECT ADVERSE REACTION REPORT

I. REACTION INFORMATION

1. Subject initials / number	1a. Country	2. Date of birth Day | Month | Year			2a. Age Years	3. Sex F/M	4-6 Reaction onset Day | Month | Year			8-12. Check all appropriate to adverse reaction
/	Denmark									☐ Patient died

7+13. Describe reaction(s) and identify reactions that are considered serious. Comment on relatedness and expectedness, intensity, outcome, whether vaccinations were discontinued.

- ☐ Patient died
- ☐ Involved or prolonged inpatient hospitalisation
- ☐ Involved persistent or significant disability or incapacity
- ☐ Life threatening
- ☐ Other medically important condition

II. SUSPECT DRUG(S) INFORMATION

14. Suspect drug(s) name(s) and batch number(s)

15. Daily dose given of suspect drug(s)

16. Route(s) and sites of administration

17. Indication(s) for use

Diagnosis of Tuberculosis

18. Date(s) of administration of suspect drug(s)

19. Therapy duration

N/A

20. Did reaction abate after stopping drug?

☐ yes ☐ no ☒ NA

21. Did reaction reappear after reintroduction?

☐ yes ☐ no ☒ NA

III. CONCOMITANT DRUG(S) AND HISTORY

22. List relevant past drug history and concomitant drug(s) and dates of administration

23. Other relevant medical history and concurrent conditions.

IV. MANUFACTURER INFORMATION

24a. Name and address of manufacturer

Statens Serum Institut, Artillerivej 5, DK-2300 Copenhagen S, Denmark

24b. Manufacturer Control No.

24c. Date received by manufacturer

24d. Report source

☒ study ☐ literature

☐ health professional

Date and signature of initial reporter:

Print name
of investigator:

Date of this report

25a. Report type

☐ initial ☐ follow-up

Date and signature of reporter of this report:

Print name
of reporter:

Use this page if you have further comments or if additional space is needed for any of the fields of the CIOMS FORM. Please state No. of the field you continue on this page.

Field No.	Comments or continuation of text
1.	*This field should always be completed:*<br>Fill in Subject initials again:<br>Fill in Subject number again:

WORLD MEDICAL ASSOCIATION DECLARATION OF HELSINKI
Ethical Principles for Medical Research Involving Human Subject

Adopted by the 18th WMA General Assembly, Helsinki, Finland, June 1964, and amended by the
29th WMA General Assembly, Tokyo, Japan, October 1975
35th WMA General Assembly, Venice, Italy, October 1983
41st WMA General Assembly, Hong Kong, September 1989
48th WMA General Assembly, Somerset West, Republic of South Africa, October 1996
and the 52nd WMA General Assembly, Edinburgh, Scotland, October 2000
Note of Clarification on Paragraph 29 added by the WMA General Assembly, Washington 2002
Note of Clarification on Paragraph 30 added by the WMA General Assembly, Tokyo 2004

A. INTRODUCTION

1. The World Medical Association has developed the Declaration of Helsinki as a statement of ethical principles to provide guidance to physicians and other participants in medical research involving human subjects. Medical research involving human subjects includes research on identifiable human material or identifiable data.

2. It is the duty of the physician to promote and safeguard the health of the people. The physician's knowledge and conscience are dedicated to the fulfillment of this duty.

3. The Declaration of Geneva of the World Medical Association binds the physician with the words, "The health of my patient will be my first consideration," and the International Code of Medical Ethics declares that, "A physician shall act only in the patient's interest when providing medical care which might have the effect of weakening the physical and mental condition of the patient."

4. Medical progress is based on research which ultimately must rest in part on experimentation involving human subjects.

5. In medical research on human subjects, considerations related to the well-being of the human subject should take precedence over the interests of science and society.

6. The primary purpose of medical research involving human subjects is to improve prophylactic, diagnostic and therapeutic procedures and the understanding of the aetiology and pathogenesis of disease. Even the best proven prophylactic, diagnostic, and therapeutic methods must continuously be challenged through research for their effectiveness, efficiency, accessibility and quality.

7. In current medical practice and in medical research, most prophylactic, diagnostic and therapeutic procedures involve risks and burdens.

8. Medical research is subject to ethical standards that promote respect for all human beings and protect their health and rights. Some research populations are vulnerable and need special protection. The particular needs of the economically and medically disadvantaged must be recognized. Special attention is also required for those who cannot give or refuse consent for themselves, for those who may be subject to giving consent under duress, for those who will not benefit personally from the research and for those for whom the research is combined with care.

9. Research Investigators should be aware of the ethical, legal and regulatory requirements for research on human subjects in their own countries as well as applicable international requirements. No national ethical, legal or regulatory requirement should be allowed to reduce or eliminate any of the protections for human subjects set forth in this Declaration.

B. BASIC PRINCIPLES FOR ALL MEDICAL RESEARCH

1. It is the duty of the physician in medical research to protect the life, health, privacy, and dignity of the human subject.

2. Medical research involving human subjects must conform to generally accepted scientific principles, be based on a thorough knowledge of the scientific literature, other relevant sources of information, and on adequate laboratory and, where appropriate, animal experimentation.

3. Appropriate caution must be exercised in the conduct of research which may affect the environment, and the welfare of animals used for research must be respected.

4. The design and performance of each experimental procedure involving human subjects should be clearly formulated in an experimental protocol. This protocol should be submitted for consideration, comment, guidance, and where appropriate, approval to a specially appointed ethical review committee, which must be independent of the investigator, the sponsor or any other kind of undue influence. This independent committee should be in conformity with the laws and regulations of the country in which the research experiment is performed. The committee has the right to monitor ongoing trials. The researcher has the obligation to provide monitoring information to the committee, especially any serious adverse events. The researcher should also submit to the committee, for review, information regarding funding, sponsors, institutional affiliations, other potential conflicts of interest and incentives for subjects.

5. The research protocol should always contain a statement of the ethical considerations involved and should indicate that there is compliance with the principles enunciated in this Declaration.

6. Medical research involving human subjects should be conducted only by scientifically qualified persons and under the supervision of a clinically competent medical person. The responsibility for the human subject must always rest with a medically qualified person and never rest on the subject of the research, even though the subject has given consent.

7. Every medical research project involving human subjects should be preceded by careful assessment of predictable risks and burdens in comparison with foreseeable benefits to the subject or to others. This does not preclude the participation of healthy volunteers in medical research. The design of all studies should be publicly available.

8. Physicians should abstain from engaging in research projects involving human subjects unless

they are confident that the risks involved have been adequately assessed and can be satisfactorily managed. Physicians should cease any investigation if the risks are found to outweigh the potential benefits or if there is conclusive proof of positive and beneficial results.Medical research involving human subjects should only be conducted if the importance of the objective outweighs the inherent risks and burdens to the subject. This is especially important when the human subjects are healthy volunteers.

9. Medical research is only justified if there is a reasonable likelihood that the populations in which the research is carried out stand to benefit from the results of the research.

10. The subjects must be volunteers and informed participants in the research project.

11. The right of research subjects to safeguard their integrity must always be respected. Every precaution should be taken to respect the privacy of the subject, the confidentiality of the patient's information and to minimize the impact of the study on the subject's physical and mental integrity and on the personality of the subject.

12. In any research on human beings, each potential subject must be adequately informed of the aims, methods, sources of funding, any possible conflicts of interest, institutional affiliations of the researcher, the anticipated benefits and potential risks of the study and the discomfort it may entail. The subject should be informed of the right to abstain from participation in the study or to withdraw consent to participate at any time without reprisal. After ensuring that the subject has understood the information, the physician should then obtain the subject's freely-given informed consent, preferably in writing. If the consent cannot be obtained in writing, the non-written consent must be formally documented and witnessed.

13. When obtaining informed consent for the research project the physician should be particularly cautious if the subject is in a dependent relationship with the physician or may consent under duress. In that case the informed consent should be obtained by a well-informed physician who is not engaged in the investigation and who is completely independent of this relationship.

14. For a research subject who is legally incompetent, physically or mentally incapable of giving consent or is a legally incompetent minor, the investigator must obtain informed consent from the legally authorized representative in accordance with applicable law. These groups should not be included in research unless the research is necessary to promote the health of the population represented and this research cannot instead be performed on legally competent persons.

15. When a subject deemed legally incompetent, such as a minor child, is able to give assent to decisions about participation in research, the investigator must obtain that assent in addition to the consent of the legally authorized representative.

16. Research on individuals from whom it is not possible to obtain consent, including proxy or advance consent, should be done only if the physical/mental condition that prevents obtaining informed consent is a necessary characteristic of the research population. The specific reasons for involving research subjects with a condition that renders them unable to give informed consent should be stated in the experimental protocol for consideration and approval of the review committee. The protocol should state that consent to remain in the research should be obtained as soon as possible from the individual or a legally authorized surrogate.

17. Both authors and publishers have ethical obligations. In publication of the results of research, the investigators are obliged to preserve the accuracy of the results. Negative as well as positive results should be published or otherwise publicly available. Sources of funding, institutional affiliations and any possible conflicts of interest should be declared in the publication. Reports of experimentation not in accordance with the principles laid down in this Declaration should not be accepted for publication.

C. ADDITIONAL PRINCIPLES FOR MEDICAL RESEARCH COMBINED WITH MEDICAL CARE

1. The physician may combine medical research with medical care, only to the extent that the research is justified by its potential prophylactic, diagnostic or therapeutic value. When medical research is combined with medical care, additional standards apply to protect the patients who are research subjects.

2. The benefits, risks, burdens and effectiveness of a new method should be tested against those of the best current prophylactic, diagnostic, and therapeutic methods. This does not exclude the use of placebo, or no treatment, in studies where no proven prophylactic, diagnostic or therapeutic method exists. See footnote

3. At the conclusion of the study, every patient entered into the study should be assured of access to the best proven prophylactic, diagnostic and therapeutic methods identified by the study. See footnote

4. The physician should fully inform the patient which aspects of the care are related to the research. The refusal of a patient to participate in a study must never interfere with the patient-physician relationship.

   In the treatment of a patient, where proven prophylactic, diagnostic and therapeutic methods do not exist or have been ineffective, the physician, with informed consent from the patient, must be free to use unproven or new prophylactic, diagnostic and therapeutic measures, if in the physician's judgement it offers hope of saving life, re-establishing health or alleviating suffering. Where possible, these measures should be made the object of research, designed to evaluate their safety and efficacy. In all cases, new information should be recorded and, where appropriate, published. The other relevant guidelines of this Declaration should be followed.

TESEC-01

Deltagerinformation om deltagelse i et videnskabeligt forsøg

STATENS SERUM INSTITUT

1 Titel

" Et fase 1 klinisk forsøg i raske voksne for at undersøge, om et nyt diagnostisk hudtest reagens (rdESAT6 + rCFP-10) til diagnostik af tuberkulose er sikkert at anvende i forskellige doser samt om der opstår en hudreaktion med rødme og hævelse (sensibilisering) efter to på hinanden følgende injektioner ved 'Mantoux' metoden".

2 Indledning og formål

Dette er en forespørgsel om deltagelse i et videnskabeligt forsøg som Statens Serum Institut (SSI) har taget initiativ til at gennemføre. SSI har udviklet et forbedret hudtest reagens til diagnosticeringen af tuberkulosesmitte, som led i bekæmpelsen af denne sygdom.

Det nuværende hudtestreagens (tuberkulin, PPD) har været brugt i hele verden gennem de sidste 100 år som en støtte i forbindelse med diagnosticeringen af tuberkulose. Hudtestreagenset sprøjtes ind lige under huden og i tilfælde af en tuberkulose infektion, udvikler personen rødme og hævelse på injektionsstedet. Raske personer reagerer ikke. Den nuværende tuberkulin test har dog flere ulemper, idet den også kan give lokal rødme og hævelse, hvis en person er vaccineret mod tuberkulose med Calmette vaccinen (BCG-vaccinen - den eneste nuværende tuberkulose vaccine til mennesker) eller har en infektion med ufarlige mykobakterier beslægtet med tuberkulosebakterien.

Det nye hudtestreagens er mere specifikt. Det indeholder en opløsning af proteinerne rdESAT-6 + rCFP-10, som hovedsageligt findes i tuberkulosebakterien samt i små mængder i tuberkulin testen, men hverken i BCG vaccinen eller i de fleste ufarlige mykobakterier i omgivelserne.

Forsøget skal undersøge forekomsten af bivirkninger i raske forsøgspersoner efter én eller to injektioner af rdESAT-6 + rCFP-10 samt om der fremkommer en lokal reaktion med rødme og hævelse på injektionsstedet efter to injektioner af rdESAT-6 + rCFP-10 (sensibilisering). Denne deltagerinformation giver dig information omkring selve forsøget og om hvad din eventuelle deltagelse vil indebære. Brug den tid du har brug for til at læse informationen og spørg endelig, hvis der er noget, du bliver i tvivl om.

Tuberkulose er fortsat en af de sygdomme, der på verdensplan forårsager stor sygelighed og dødelighed med op til 9 millioner nye tilfælde og 2 millioner dødsfald årligt. Sygdommen forårsages af infektion med tuberkulosebakterien *"Mycobacterium tuberculosis"*.

Det ene protein i det nye hudtestreagens (rdESAT-6) blev for første gang afprøvet i mennesker i 2005/2006 i et klinisk fase 1a forsøg i Holland. Reagenset viste sig at være sikkert og det kunne effektivt skelne raske forsøgsdeltagere fra forsøgsdeltagere, som tidligere havde været i behandling for tuberkulose. Kun personer behandlet for tuberkulose fik en lokal hudtestreaktion med rødme og hævelse.

I 2007 viste et fase 1b klinisk forsøg på Rigshospitalet med raske forsøgspersoner, at to injektioner af rdESAT-6 givet med et interval på 1 måned i visse tilfælde kunne give en lokal reaktion med rødme og hævelse (sensibilisering) svarende til den reaktion der forventes hvis personen havde været smittet med tuberkulose. Forsøget viste endvidere at risikoen for en sensibiliseringsreaktion kunne begrænses, hvis tidsintervallet mellem de to doser blev øget.

For bedre at kunne identificere tuberkuloseinfektioner har SSI i dag valgt at kombinere rdESAT-6 + rCFP-10 i en diagnostisk hudtest for at øge testens følsomhed.

I videnskabelige forsøg i rotter er der ikke observeret bivirkninger efter 4 injektioner af 10 mikrogram rdESAT-6 + rCFP-10 givet med et interval på 1 uge (svarende til en 10 gange højere dosis end den højeste dosis der skal gives i det planlagte kliniske forsøg).

I et videnskabeligt forsøg med marsvin blev det vist at to injektioner af 1 mikrogram rdESAT-6 + rCFP-10 givet med et interval på én måned gav lokal rødme på injektionsstedet svarende til en sensibiliseringsreaktion.

I det planlagte kliniske forsøg vil **50 raske frivillige mellem 18 og 55 år** tilfældigt blive inddelt i 5 grupper à 10 personer (gruppe A, B, C, D og E).

Forsøgsdeltagerne i gruppe A, B, C og D gives to injektioner lige under huden på 0,1 mL med en dosis på 0.01 (gruppe A og B) eller 0.1 (gruppe C og D) mikrogram rdESAT-6 + rCFP-10 med et tidsinterval på enten 6 uger (gruppe A og C) eller 12 uger (gruppe B og D). Forsøgsdeltagere i grupperne A, B, C og D vil skulle møde til i alt 6 besøg.

Forsøgsdeltagerne i gruppe E gives én enkelt injektion på 0.1 mL lige under huden med en dosis på 1.0 mikrogram rdESAT-6 + rCFP-10. Forsøgsdeltagere i gruppe E vil skulle møde til i alt 4 besøg.

Dette videnskabelige forsøg vil blive udført på Epidemiklinikken afd. M på Rigshospitalet i København. Forsøget er godkendt af den danske lægemiddelstyrelse samt de videnskabsetiske komiteer for Region Hovedstaden. (Journalnumre vil blive indsat).

3 Generel deltager information

Det er frivilligt at deltage i forsøget og du kan til enhver tid vælge at træde ud af forsøget uden begrundelse også selvom samtykke- og fuldmagtserklæringen er underskrevet. Du kan kun deltage hvis du både har modtaget mundtlig information om forsøget, har læst den skriftlige information og efterfølgende har underskrevet samtykke- og fuldmagtserklæringen. Du har ret til at medbringe en bisidder ved

modtagelsen af den mundtlige information, og du har ret til 24 timers betænkningstid efter at have modtaget informationen omkring forsøget, inden du beslutter dig.

Hvis du beslutter dig for at deltage i dette forsøg vil forsøgets in- og eksklusions kriterier blive gennemgået med dig. Disse inkluderer oplysninger omkring dit generelle helbred, eventuelle sygdomme og eventuel kontakt med tuberkulose patienter. Under forsøget vil rutineret personale tage blod- og urinprøver for at undersøge om du er helt rask samt blodprøver for at undersøge hvordan dit immunforsvar reagerer på ESAT-6 + CFP-10 (såkaldt QuantiFERON analyse). I alt vil du skulle afgive 2- 3 blodprøver (afhængig af hvilken forsøgsgruppe du kommer i) og 2 urinprøver svarende til, at der vil blive trukket max. 45 ml blod alt i alt under forsøget. Digitale billeder af indstiksstedet for rdESAT-6 +rCFP-10 hudtesten vil blive taget 2-3 gange i løbet af forsøget.

Det er en forudsætning for deltagelse i forsøget, at du er rask og velfungerende. Derfor vil en blodprøve blive taget for at undersøge for HIV (human immundefekt virus). Før denne prøve tages vil en erfaren sygeplejerske eller læge give dig de nødvendige oplysninger samt efterfølgende give dig svaret. Undersøgelse for Hepatitis B og C (leverbetændelse) vil også blive udført før evt. deltagelse i forsøget. Alle resultater vil blive behandlet fortroligt og hvis resultaterne af disse undersøgelser viser sig positive vil du blive vejledt af en læge tilknyttet forsøget. Er du kvinde vil graviditetsundersøgelser (urinprøver) blive udført 2- 3 gange i løbet af forsøget (afhængig af hvilken forsøgsgruppe du kommer i), for at sikre, at du ikke er gravid. En lægeundersøgelse udføres 2 gange på alle forsøgsdeltagere i løbet af forsøget.

Oplysninger omkring dine personlige data, helbredsmæssige forhold og andre fortrolige oplysninger som måtte komme frem i forbindelse med forsøget er omfattet af tavshedspligt. Du vil forblive anonym i alle forsøgets faser og dine data vil beskyttes efter lov om behandling af personoplysninger samt sundhedsloven. Adgang til forsøgsdokumenterne samt blodprøver vil blive kontrolleret af autoriseret personale tilknyttet forsøget, og data vil blive opbevaret i henhold til gældende love og retningslinier. Data fra forsøget vil blive opbevaret i 15 år. Ved at underskrive samtykke- og fuldmagtserklæringen giver du autoriseret personale tilknyttet forsøget (hospitalspersonale samt personale fra Statens Serum Institut), repræsentanter fra de danske myndigheder samt repræsentanter fra den etiske komité adgang til forsøgsdokumenterne. Blodprøverne vil ikke blive opbevaret efter, at de anførte analyser er gennemført.

4 Mulige bivirkninger, risici, komplikationer og ulemper

Det ene protein i det nye hudtestreagens (rdESAT-6) er som før nævnt afprøvet i to kliniske forsøg i mennesker i henholdsvis Holland og Danmark.

I begge kliniske forsøg var hudtestreagenset sikkert og ingen alvorlige bivirkninger blev observeret.

Forsøget i Holland viste at rdESAT-6 hudtestreagens kunne skelne mellem raske forsøgsdeltagere og forsøgsdeltagere, der tidligere havde været i behandling for tuberkulose.

I forsøget i Danmark, hvor kun raske blev testet, oplevede én deltager lokal hævelse og rødme efter to på hinanden følgende injektion af rdESAT-6 med 4 ugers mellemrum.

Dette kliniske forsøg har til formål at undersøge forekomsten af eventuelle bivirkninger i raske forsøgspersoner efter én eller to injektioner af det nye hudtestreagens rdESAT-6 + rCFP-10 samt om der fremkommer en lokal reaktion med rødme og hævelse på injektionsstedet (sensibilisering) efter to injektioner. Da forekomsten af disse lokale symptomer menes at være knyttet til tidsintervallet mellem to doser vil to tidsintervaller på 6 og 12 uger mellem doserne blive afprøvet i dette kliniske forsøg

I videnskabelige forsøg med rotter blev det nye hudtest reagens rdESAT-6 + rCFP-10 givet 4 gange i doser på 10 mikrogram rdESAT-6 + rCFP-10 med en uges mellemrum uden at der opstod bivirkninger. Dette svarer til en 10 gange højere dosis end den højeste dosis gruppe (gruppe E) vil blive givet i dette kliniske forsøg.

I et videnskabeligt forsøg i marsvin blev det vist at to injektioner af 1 mikrogram rdESAT-6 + rCFP-10 givet med et interval på 1 måned gav lokal rødme på injektionsstedet svarende til en eventuel sensibilisering.

I yderst sjældne tilfælde kan der opstå en akut allergisk reaktion som følge af injektionerne. I tilfælde af en sådan allergisk reaktion vil der ofte skulle indgives adrenalin eller anden medicin omgående. Denne allergiske reaktion vil altid ske umiddelbart efter injektionen, og derfor skal alle forsøgsdeltagere af sikkerhedsårsager blive til observation på Rigshospitalet 1 time efter hver indgift af rdESAT-6 + rCFP-10.

Som samlet konklusion på ovenstående vurderes det, at du i dette kliniske forsøg primært risikerer at opleve lokale reaktioner omkring indstiksstedet i form af rødme og hævelse. Der kan imidlertid opstå uforudsigelige bivirkninger som ved deltagelse i alle videnskabelige forsøg. I tilfælde af at rdESAT-6 + rCFP-10 hudtesten virker sensibiliserende/giver lokale "overfølsomhedssymptomer" vil vi bede dig om at komme ind til et kontrolbesøg 6 måneder efter dit sidste besøg i dette forsøg.

Graviditet:
Der er ikke lavet studier, der viser hvordan den nye hudtest evt. påvirker et ufødt barn, og derfor er der en teoretisk mulighed for, at hudtesten kan påvirke et ufødt barn. Kvinder, der er gravide, forventer at blive gravide eller ammer kan derfor ikke deltage i dette fase 1 kliniske forsøg. Kvinder der deltager i dette forsøg skal anvende sikker antikonception (fx p-piller, spiral eller lign.) i forsøgsperioden.

Procedurer:
At få taget en blodprøve kan i nogle tilfælde give blå mærker eller ømhed omkring indstiksstedet. I sjældne tilfælde kan blodåren, hvor prøven trækkes fra blokeres, eller der kan komme milde skader på nerverne, som kan give følelsesløshed og ømhed. Normalt forsvinder disse gener efter kort tid.

5 Fordele for deltagere

Du vil ikke opnå nogen direkte fordel af at deltage i dette forsøg, men din deltagelse har betydning for den senere vurdering af, om rdESAT-6 + rCFP-10 hudtest reagenset

kan blive et betydningsfuldt diagnostisk redskab, som kan anvendes i den fortsatte bekæmpelse af tuberkulose på verdensplan.

6 Afbrydelse af forsøget og udelukkelse fra forsøget

En generel afbrydelse af forsøget kan forekomme og vil i så fald føre til, at de enkelte deltagere skal udgå af forsøget før tid, også selvom det er imod den enkelte deltagers eget ønske at udgå. Da deltagelse i forsøget er forbundet med en meget lille risiko, og forsøget er af kort varighed, anses en generel afbrydelse dog som usandsynlig.

Den ansvarlige læge kan ligeledes til enhver tid vurdere, at en bestemt forsøgsdeltager skal udgå af forsøget, f.eks. hvis der opstår en alvorlig uønsket hændelse, eller hvis en forsøgsdeltager af andre grunde ikke længere følger forsøgsplanen.

7 Økonomi

Dette forsøg finansieres af Statens Serum Institut (SSI). SSI betaler for ansættelsen af de læger og sygeplejersker på Rigshospitalet, der i den givne periode skal være med til at udføre forsøget. Pengene vil blive overført til en særlig konto på Rigshospitalet. Overlæge Åse Bengård Andersen, Rigshospitalet er overordnet ansvarlig for forsøget. Åse Bengård Andersen har tidligere været ansat på SSI, men er nu ansat på Rigshospitalet og modtager ikke løn fra SSI.

Der vil ikke på anden måde blive udbetalt honorarer eller givet gaver fra SSI til forsøgsansvarlige på Rigshospitalet. Disse forhold vil blive beskrevet i en kontrakt, som vil blive indgået mellem SSI og Rigshospitalet, før forsøget starter.

Forsøgsdeltagere i grupperne A, B, C og D vil skulle møde til 6 besøg á ca. 60 min. varighed over en periode på 12 – 18 uger. Deltagelse i forsøget honoreres med kr. 1.800 pr. person samt transport godtgørelse på kr. 200 per besøg.

Forsøgsdeltagere tildelt gruppe E vil skulle møde til 4 besøg á ca. 60 min. varighed over en periode på 6 uger. Deltagelse i forsøget honoreres med kr. 1.200 pr. person samt transport godtgørelse på kr. 200 per gang.

Alle beløb er skattepligtige.

8 Forsikring

Hvis du oplever at få uforudsete bivirkninger af forsøgsmedicinen og forsøgsmedicinen er givet I henhold til forsøgsprotokollen, så vil du være dækket af Statens Serum Instituts produktansvarsforsikring, som er tegnet gennem "New Hampshire Insurance Company". Desuden vil du være dækket af "lov om patientforsikringen" nr. 1097 12 december 2003.

9 Kontaktperson

Åse Bengård Andersen
Epidemiklinik M 51-3-2
Rigshospitalet
Blegdamsvej 9
2100 København Ø
Tel: (vil blive annonceret)

Åse Bengård Andersen besvarer gerne spørgsmål, der måtte opstå i forbindelse med dette forsøge. Træffes bedst mellem kl… og kl…

10 Forsøgsresultater

Resultaterne fra dette videnskabelige forsøg, såvel positive som negative, vil blive offentliggjort så hurtigt, som det er muligt og fagligt forsvarligt.

Tak fordi du har taget dig tid til at læse denne deltagerinformation.
Hvis du beslutter dig for at deltage i forsøget vil du få en kopi af denne information samt en kopi af den underskrevne samtykke- og fuldmagtserklæring

Vedlagt: "Forsøgspersonens rettigheder i et biomedicinsk forskningsprojekt"

DEN CENTRALE
VIDENSKABSETISKE
KOMITÉ

The Danish National Committee
on Biomedical Research Ethics

Forsøgspersonens rettigheder i et biomedicinsk forskningsprojekt.

Som deltager i et biomedicinsk forskningsprojekt skal du vide at:

- din deltagelse i forskningsprojektet er helt frivillig og kan kun ske efter, at du har fået både skriftlig og mundtlig information om forskningsprojektet og underskrevet samtykkeerklæringen
- du til enhver tid mundtligt, skriftligt eller ved anden klar tilkendegivelse kan trække dit samtykke til deltagelse tilbage og udtræde af forskningsprojektet. Såfremt du trækker dit samtykke tilbage påvirker dette ikke din ret til nuværende eller fremtidig behandling eller andre rettigheder, som du måtte have
- du har ret til at tage et familiemedlem, en ven eller en bekendt med til informationssamtalen
- du har ret til betænkningstid, før du underskriver samtykkeerklæringen
- oplysninger om dine helbredsforhold, øvrige rent private forhold og andre fortrolige oplysninger om dig, som fremkommer i forbindelse med forskningsprojektet, er omfattet af tavshedspligt
- opbevaring af oplysninger om dig, herunder oplysninger i dine blodprøver og væv, sker efter reglerne i lov om behandling af personoplysninger og sundhedsloven
- der er mulighed for at få aktindsigt i forsøgsprotokoller efter offentlighedslovens bestemmelser. Det vil sige, at du kan få adgang til at se alle papirer vedrørende din deltagelse i forsøget, bortset fra de dele, som indeholder forretningshemmeligheder eller fortrolige oplysninger om andre
- der er mulighed for at klage og få erstatning efter reglerne i lov om klage- og erstatningsadgang inden for sundhedsvæsenet

(Dette tillæg udgives af Den Centrale Videnskabsetiske komité og kan vedhæftes den skriftlige information om det biomedicinske forskningsprojekt. Spørgsmål til et projekt skal rettes til den regionale komité, som har godkendt projektet)

SSI/Trial code: TESEC-01

Samtykke- og Fuldmagtserklæring

A. Samtykke:

Jeg har læst den skriftlige information om forskningsprojektet og fået mundtlig information i et sprog, som jeg forstår. Jeg ved nok om formålet, metoderne, fordele og ulemper til at sige ja til at deltage. Jeg er informeret om, at det er frivilligt at deltage, og at jeg når som helst og uden begrundelse kan trække mit samtykke tilbage og udtræde af forsøget.

Jeg indvilger i at deltage i forskningsprojektet og har modtaget en kopi af denne samtykkeerklæring samt en kopi af den skriftlige deltagerinformation til eget brug.

Deltagers navn (blokbogstaver): ______________________________

Dato: ________________ Underskrift: ______________________________

B. Fuldmagt:

Jeg giver hermed min **fuldmagt** til, at personer autoriseret af Lægemiddelstyrelsen, etisk komité eller Statens Serum Institut i en periode på op til 15 år efter forsøgets afslutning kan få adgang til hele min patientjournal i forbindelse med kontrol ved inspektion.

Deltagers navn (blokbogstaver): ______________________________

Dato: ________________ Underskrift: ______________________________

C. Attestation

Undertegnede forsøgsansvarlige læge eller dennes repræsentant attesterer hermed, at den skriftlige information er udleveret til forsøgspersonen, og at mundtlig information har fundet sted. Efter min overbevisning er der givet tilstrækkelig information, herunder om fordele og ulemper, til at træffe et informeret valg.
Formularen attesteres, før den udleveres.

Forsøgsansvarliges navn (blokbogstaver): ______________________________

Dato: ________________ Underskrift: ______________________________

Indemnity Statement

Trial title: An open phase I clinical trial on the safety and the risk of sensitisation by escalating doses and repeated injections of the rdESAT-6 + rCFP-10 skin test reagent following intradermal administration to healthy adults

Trial code: TESEC-01

Dear Dr. Åse Bengård Andersen,

You have kindly agreed to consider undertaking the above-mentioned clinical trial as investigator, in accordance with the protocol for the trial TESEC-01.

In the event that any recruited subject in the trial should suffer any personal injury resulting from the clinical trial, SSI agrees to indemnify the institution where the clinical trial is being undertaken, Rigshospitalet, Denmark, and, through Rigshospitalet, any of its employees or agents participating in the trial, against liability imposed by law, but not assumed voluntarily, and arising from the use of the test products in the trial, PROVIDED THAT:

1) SSI shall not indemnify against, nor have any obligation whatsoever as regards liability arising from or related to any error, omission, intentional wrongful act, or other negligence on the part of said institutions or persons, such as medical malpractice; and

2) Any such institution or person seeking indemnity
   a) has fully complied with the protocol for the trial, and
   b) has promptly notified SSI of any notice of any type of claim, or the likelihood of a claim, relating to the trial,
   c) as regards any claim, makes no statement, takes no action, nor makes any commitment affecting SSI's interests, without SSI's prior written consent, and further, provides all reasonable and necessary assistance to SSI in the defence of any claim, allowing SSI, at its cost and in its discretion, to take over the defence of any action and to have full control in handling the claim.

Please note that this letter is not a legal contract itself, but rather summarizes the main points of SSI's liability under its agreement with Rigshospitalet.

Yours sincerely,
Statens Serum Institut

Ingrid Kromann
Head of the Department of Vaccine Development

[redacted]

AIG

CERTIFICATE OF INSURANCE

This is to certify that a Policy of Insurance as described below has been issued to the Policy Holder and the coverage thereby is as follows:

**Policy Number:**	20001156
**Policy Holder:**	Statens Seruminstitut and/or subsidiary and/or associated and/or Affiliated companies
**Type of Insurance:**	Public and Products Liability
**Period of Indemnity:**	From 1st June 2008 to 31st May 2009 both days inclusive
**Limits of Liability:**	D.Kr.60,000,000 Combined Single Limit Personal Injury and Property Damage<br><br>D.Kr.60,000,000 Total Annual Aggregate for Products Liability
**Retro Date:**	1st June 1992
**SIR (Self Insured: Retention)**	(a) United States of America:<br>D.Kr.5,000,000 per Occurrence<br>(b) Elsewhere in the world:<br>D.Kr.1,500,000 per Occurrence
**Wording:**	GAP claims made form (amended)
**Principal Operations:**	All operations of the Policy Holder including but not limited to:<br>(a) the provision of diagnostic services<br>(b) the manufacture sale and distribution of vaccines and other pharmaceutical products including blood products diagnostic agents and culture media.<br>(c) Research and development

(d) Property owners

Exclusions: Usual Policy exclusions plus

(a) exclusion of efficacy

(b) total pollution exclusion in respect of United States of America

Extensions: (a) Blanket Contractual Liability

(b) Including Clinical Trials Coverage

Nothing herein contained shall in any way be held or construed to vary, alter or waive any of the terms, conditions or provisions of the Policy.

SIGNED FOR AND ON BEHALF OF THE COMPANY

AIG UK LIMITED

The Policy document is only summarised by this Certificate.
For full information refer to the Policy.

AIG UK Limited
This insurance is underwritten by AIG UK Limited which is authorised and regulated by the Financial Services Authority (FSA number 202628). This information can be checked by visiting the FSA website (www.fsa.gov.uk/register). AIG UK Limited is a member of the Association of British Insurers and a member company of American International Group, Inc. Registered in England: company number 1486260. Registered address: The AIG Building, 58 Fenchurch Street, London, EC3M 4AB.

Mantoux testing technique

TUBERCULIN PPD RT 23 SSI

STATENS
SERUM
INSTITUT

prevention and control of infectious diseases and congenital disorders

Guidelines for Mantoux skin testing

Tuberculin PPD RT 23 SSI 2 T.U. is a premixed, ready to use solution for Mantoux skin test to assist in clinical diagnosis of tuberculosis.
PPD RT 23 SSI is also used as a standard tuberculin in skin test surveys to determine the prevalence of Mycobacterium tuberculosis *in a community.*

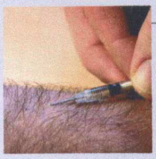

Dosage and injection site

- The most common strength for diagnostic purposes is 2 T.U. Tuberculin PPD RT 23 SSI. The dosage is always 0.1 ml.
- The dosage should be given strictly intradermally (intracutaneously) in the middle third of the forearm, using a 1.0 ml graduated syringe fitted with a short bevel needle, 25 or 26 gauge.
- Fill the syringe immediately before use.
- Draw up slightly more than 0.1 ml of PPD RT 23 SSI, remove any air bubbles, and reduce the volume to exactly 0.1 ml.

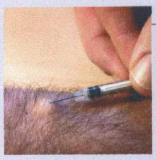

Injection technique

- Use separate sterile needles and syringes for each patient.
- Penetrate the skin with the needle bevel up, entering just the superficial layer of the skin. The needle should be visible through the epidermis during insertion.
- Inject the solution slowly and a small papule of 8 - 10 mm in diameter will appear and remain for about 10 minutes.
- If no papule is formed, the solution might have been injected too deeply, and the skin test should be repeated at another site, at least 4 or more centimetres away from the first injection.

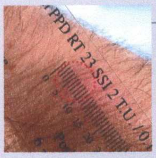

Evaluating the reaction

- The result should be evaluated approximately 3 days (72 hours) after the injection.
- A positive reaction to Tuberculin PPD RT 23 SSI is defined as a flat, uneven, slightly raised induration with a diameter of at least 6 millimetres, surrounded by a more or less defined area of redness.
- Only the induration should be assessed.

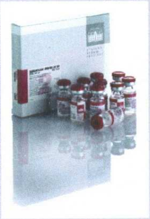

How to read the Mantoux test<br>Diameter of induration		
Negative<br>0 - 5 mm	Positive<br>6 - 14 mm	Strongly positive<br>15+ mm

Reactions with a diameter larger than 15 millimetres are defined as strongly positive and give a strong indication of infection with *Mycobacterium tuberculosis* complex.
Previous vaccination with BCG or exposure to non tuberculous mycobacteria can give a positive reading due to cross reaction with common mycobacterial antigens.

Statens Serum Institut
Artillerivej 5
2300 Copenhagen S
Denmark

Tel.: +45 3268 3918
Fax: +45 3268 3167
export.dept@ssi.dk
www.ssi.dk

51964 • SSI/09.03 • KROGH DK

Den Centrale Videnskabs-
etiske Komité

Før du beslutter dig

Forsøgspersoner til
sundhedsvidenskabelige forsøg

De Videnskabsetiske
Komitéer i Danmark

FØR DU BESLUTTER DIG

- Forsøgspersoner til sundhedsvidenskabelige forsøg

I denne folder finder du en række oplysninger, som du bør kende til, når du skal tage stilling til at deltage i et sundhedsvidenskabeligt forsøg.

Folderen sætter fokus på dine rettigheder som forsøgsperson og på den information, du har krav på at modtage inden deltagelse i forsøg.

Du kan desuden finde oplysninger om den praktiske tilrettelæggelse af forsøg og om de videnskabsetiske komitéer, der i Danmark godkender alle sundhedsvidenskabelige forsøg med mennesker.

DELTAGELSE I FORSØG

Når du kommer i kontakt med sundhedsvæsenet, kan du blive spurgt, om du vil deltage i et videnskabeligt forsøg.

Du kan blive spurgt, om du vil deltage i en afprøvning af en ny medicin eller operationsmetode, eller du kan blive spurgt om, du vil medvirke til, at der foretages undersøgelser, udover hvad man normalt ville gøre hos patienter i din situation.

Som et led i forskningen er det nødvendigt at udføre forsøg på mennesker, hvis vi ønsker at opnå ny viden om sygdom, og hvis vi vil blive dygtigere til at behandle sygdomme.

Det er frivilligt at deltage i forsøg, og hvis du vælger at deltage, er der i lovgivningen fastsat regler for, hvordan og på hvilke betingelser forsøg må gennemføres. Formålet med lovgivningen er at sikre, at forsøgspersonerne ikke lider skade.

Deltagere skal have en information som i et letforståeligt sprog giver oplysninger om, hvad forsøget går ud på, og hvilke konsekvenser det kan få for forsøgspersonen.

PLANLÆGNING AF FORSØGET

Den læge eller forsker, der ønsker at gennemføre et forsøg, udarbejder i første omgang en beskrivelse af formålet med forsøget og en plan for, hvordan forsøget skal gennemføres.

Forskeren skal beskrive, hvilken risiko der er forbundet med forsøget, og om der kan opstå bivirkninger for de mennesker, der deltager.

Deltagere skal have både mundtlig og skriftlig information som i et letforståeligt sprog giver oplysninger om, hvad forsøget går ud på, og hvilke fordele og ulemper det kan få for forsøgspersonen.

Forsøget skal godkendes af en videnskabsetisk komité, før det kan sættes i gang. Det betyder, at også informationsmateriale skal godkendes af en videnskabsetisk komité. Hvis forsøget handler om afprøvning af medicin, skal det desuden godkendes af Lægemiddelstyrelsen.

I hvert enkelt tilfælde vurderer komitéen nøje, hvilken risiko og eventuel nytteværdi, et forsøg kan indebære for forsøgspersonerne. Hvis et forsøg på nogen måde er uforsvarligt at gennemføre, bliver det ikke godkendt.

VIGTIGT AT VIDE

Inden du beslutter dig til, om du vil deltage, skal du have en klar mundtlig og skriftlig information om forsøget. Hvis du ønsker at deltage, skal du skrive under på en erklæring om, at du har fået information om forsøget, og at du er villig til at deltage.

Dine rettigheder:

- du har krav på fyldestgørende information om forsøget.
- du har ret til at tage et familiemedlem, en ven eller en bekendt med, når du modtager informationen,
- du har ret til betænkningstid, så vidt muligt 24 timer, før du beslutter dig,
- din underskrift bekræfter, at du har givet tilsagn til at deltage,
- du kan til enhver tid og uden begrundelse trække dit tilsagn tilbage og træde ud af forsøget,
- hvis du trækker dit tilsagn tilbage, må dette ikke have nogen negativ virkning for dig - Det vil sige, at du har ret til samme behandling som en patient, der ikke deltager i forsøget,
- fortrolige oplysninger om dig i relation til forsøget er omfattet af tavshedspligt,
- du har mulighed for at få aktindsigt. Det vil sige, at du kan få adgang til at se alle papirer vedrørende din deltagelse i forsøget, bortset fra de dele, der indeholder forretningshemmeligheder eller private oplysninger om andre,
- du har mulighed for at klage over behandlingen og søge erstatning, hvis der opstår skader i forbindelse med forsøget,
- du har krav på at blive orienteret, hvis forsøget ændrer karakter, eller hvis der under forsøget kommer nye oplysninger frem om effekt, risiko, bivirkninger eller lignende,
- du kan bede om at blive orienteret om de resultater, der er opnået efter forsøgets afslutning,

For folk, der er umyndige, bevidsthedsslørede og lignende gælder særlige regler.

INFORMATION OM FORSØGET

Den skriftlige information skal indeholde oplysninger om følgende:

- du deltager i et sundhedsvidenskabeligt forsøg, og modtager ikke almindelig behandling,
- formålet med forsøget og hvordan det udføres,
- i tilfælde af lægemiddelafprøvninger: Information om hvilke godkendte og ikke-godkendte lægemidler der anvendes,
- information om, hvorvidt der trækkes lod mellem flere forskellige behandlingsformer, hvoraf én kan være behandling med et uvirksomt præparat,
- risici, bivirkninger og ulemper ved deltagelse i forsøget,
- nytten af forsøget,
- under hvilke omstændigheder du kan udelukkes fra forsøget,
- under hvilke omstændigheder forsøget kan blive afbrudt,
- navn, adresse og telefonnummer på forsøgets kontaktperson,
- eventuel økonomisk støtte som forskeren modtager fra private virksomheder og fonde,
- hvilken sygdomsbehandling du vil få, hvis du vælger at sige nej til at deltage i forsøget.

Den skriftlige information skal udleveres i god tid, så vidt muligt 24 timer inden du tager stilling til, om du vil deltage. Herefter mødes du med den eller de personer, der er ansvarlig for forsøget. Det er vigtigt, at du her benytter lejligheden og får den mundtlige information og stiller spørgsmål, hvis du er i tvivl.

HVIS DU VIL KLAGE

Hvis du som forsøgsperson bliver udsat for en utilfredsstillende behandling, har du mulighed for at klage.

Hvis du deltager i forsøget som patient og er i behandling, skal du i de fleste tilfælde sende din klage til Patientklagenævnet (Frederiksborggade 15, 2. 1360 Kbh. K). Hvis du ønsker at søge erstatning, skal du sende en ansøgning til Patientforsikringen (Nytorv 5, 1450 Kbh. K).

Du kan få yderligere information og de nødvendige blanketter gennem de ansvarlige for forsøget.
I tvivlstilfælde kan du rette henvendelse gennem den regionale videnskabsetiske komité, der dækker din region.

DE VIDENSKABSETISKE KOMITÈER

I Danmark har vi 8 regionale videnskabsetiske komitéer, alle med repræsentation af lægfolk og forskere. Komitésystemets primære opgave er at sikre beskyttelsen af forsøgspersoner, der deltager i sundhedsvidenskabelige forsøg, samtidig med at der skabes mulighed for udvikling af ny, værdifuld viden. Endvidere skal systemet virke for udbredelsen af kendskabet til de etiske problemstillinger, der kan være forbundet med den sundhedsvidenskabelige forskning.

I de videnskabsetiske komitéer sidder både forskere og ikke-forskere, såkaldte lægpersoner. Der er et flertal af lægpersoner i komitéerne.

Lægpersonerne skal sikre, at almindelige menneskers synspunkter bliver tilgodeset, når man vurderer det etisk forsvarlige i forsøget. Især er det en fordel, at der er ikke-forskere med til bedømmelse af patientinformationen.

De videnskabsetiske komitéer i Danmark

De Videnskabsetiske Komitéer for Region Hovedstaden (4 komitéer)
Regionsgården
Kongens Vænge 2
3400 Hillerød
Tlf. 4820 5000
Hjemmeside: www.vek-hovedstaden.dk
e-mail: vek@regionh.dk

Den Videnskabsetiske Komité for Region Sjælland (1 komité)
Alléen 15
4180 Sorø
Tlf. 5787 5255
Hjemmeside: http://www.regionsjaelland.dk/get/69412475.html
e-mail: hob@regionsjaelland.dk

Den Videnskabsetiske Komité for Region Syddanmark (1 komité)
Heden 16 st.
5000 Odense C
Tlf. 6541 3425, 6541 1909 og 6541 3973
Hjemmeside: www.ouh.dk/vidkom
e-mail: vejle-fynkomiteen@ouh.fyns-amt.dk

Den Videnskabsetiske Komité for Region Midtjylland (1 komité)
Sundhedssekretariatet
Skottenborg 26
Postboks 21
8800 Viborg
Tlf. 8728 4410, 8728 4409 og 8728 4408
Hjemmeside: http://www.rm.dk/sundhed/forskning/videnskabsetisk+komite
e-mail: komite@rm.dk

Den Videnskabsetiske Komité for Region Nordjylland (1 komité)
Niels Bohrs Vej 30
9220 Aalborg Ø
Tlf. 9635 1041
Hjemmeside:
http://www.rn.dk/SundhedOgSygehuse/Raad+og+udvalg/Videnskabsetisk+komité/
e-mail: lak@rn.dk

FLERE OPLYSNINGER

Du kan få flere oplysninger i de videnskabsetiske komitéer om det at være forsøgsperson.

Den Centrale Videnskabsetiske Komité
Slotholmsgade 12
1216 København K

Tlf.: 7226 9370
Fax: 7226 9380
Email: cvk@im.dk
Homepage:
www.cvk.im.dk
